# Unaltered maximal power and submaximal performance correlates with an oxidative *vastus lateralis* proteome phenotype during tapering in male cyclists

**DOI:** 10.14814/phy2.70302

**Published:** 2025-04-23

**Authors:** Pieter de Lange, Giuseppe Petito, Hannah L. Notbohm, Antonia Giacco, Giovanni Renzone, Elena Silvestri, Arianna Cuomo, Frank Suhr, Thorsten Schiffer, Jonas Zacher, Federica Cioffi, Rosalba Senese, Andrea Scaloni, Moritz Schumann, Wilhelm Bloch

**Affiliations:** ^1^ Department of Environmental, Biological, and Pharmaceutical Sciences and Technologies University of Campania “Luigi Vanvitelli” Caserta Italy; ^2^ Department of Molecular and Cellular Sport Medicine German Sports University Cologne Germany; ^3^ Department of Sciences and Technologies University of Sannio Benevento Italy; ^4^ Proteomics, Metabolomics and Mass Spectrometry Laboratory ISPAAM, National Research Council Portici Italy; ^5^ Division of Molecular Exercise Physiology, Faculty of Life Sciences: Food, Nutrition and Health University of Bayreuth Kulmbach Germany; ^6^ Outpatient Clinic for Sports Traumatology and Public Health Consultation German Sports University Cologne Germany; ^7^ Department of Preventative and Rehabilitative Sports and Performance Medicine, Institute of Cardiovascular Research and Sports Medicine German Sports University Cologne Germany; ^8^ Department of Sports Medicine and Exercise Therapy Chemnitz University of Technology Chemnitz Germany

**Keywords:** aerobic capacity, fiber percentage ratio, protein profile, threshold power, training intensity, training volume

## Abstract

Little is known on how a short‐term reduction of training volume changes muscle proteome and physiological parameters. We investigated the impact of halving training volume during regular training of cyclists on physiological parameters in relation to *vastus lateralis* protein profiles and fiber percentage ratios. Fifteen male cyclists (age: 30.1 ± 9.6 yrs.; VO_2_max: 59.4 ± 4.4 mL∙kg^−1^∙min^−1^; weekly training volume: 8.7 ± 2.3 h) participated in an 11‐week training intervention. During 2 weeks after a shared training programme for 9 weeks, a control group continued training and a taper group reduced training volume by 50%. No end‐point differences were found for peak power output, maximal oxygen uptake, or peak and mean power in a sprint test (*p* > 0.05), although in the taper group, muscle proteins involved in mitochondrial aerobic respiration increased whereas those involved in translation, protein catabolism, and actin organization decreased, without between‐group differences in type I/type II fiber percentage ratios. Tapering did not decrease power at the first (LT_1_) and second lactate threshold (LT_2_) compared to t0, whereas power increased in the control group (LT_1_: 216 ± 28 W vs. 238 ± 11 W, *p* = 0.042, LT_2_: 290 ± 42 W vs. 318 ± 13 W, *p* = 0.005). Our data indicate that transient 50% training volume reductions may be beneficial for oxidative metabolism in muscle.

## 1 INTRODUCTION

Tapering is characterized by a temporal alleviation of a training program and can be achieved by reducing training intensity and/or training volume (García‐Pallarés et al., 2009; Mujika & Padilla, 2000). At the physiological level, the effects of short‐term tapering are inconsistent and range from reduced cardiovascular and ventilatory function (Pedlar et al., 2018) and reduced endurance performance (García‐Pallarés et al., 2009; Madsen et al., 1993; Mujika & Padilla, 2000) on the one hand to reduced symptoms of fatigue and increased physical performance (Bosquet et al., 2007; Mujika, 2010) on the other. This observed discrepancy derives from differences in duration and mode of tapering, and maintaining intensity has been proposed to outvalue maintaining volume in view of a beneficial outcome (Spiering et al., 2021).

Of note, how tapering interventions alter the overall protein profile in human skeletal muscle has not been studied. Although different training modes and exercise timing have been shown to alter protein translation (Robinson et al., 2017; Savikj et al., 2022), information on changes in protein translation in response to reductions in training volume is scarce. One study reported reduced content of selected mitochondrial proteins and related transcription factors upon reduction of training volume in response to high‐intensity interval training (Granata et al., 2016). A second study reported reduced transcription of selected proteolytic, myogenic, and protective genes in response to halved training volumes of indoor running (Luden et al., 2010).

Taking the above into consideration, the aim of this study was to compare the effects of a two‐week 50% reduction of training volume, whilst maintaining training intensity, on cycling performance determinants and changes in *vastus lateralis* proteome profiles and fiber type ratios. By assessing protein interactomes and functionally interacting specific clusters, we explored how the training volume reduction intervention changed structural and metabolic features, and how these could be related to the physiological findings.

## 2 MATERIALS AND METHODS

### 2.1 Experimental design

The present study consisted of an 11‐week training intervention. For the first 9 weeks, all participants trained according to the same training intensity distribution (TID). Following this initial loading, participants were matched into a control (CON) and a taper group (TAPER) based on their training volume and cycling performance. During the final 2 weeks, CON continued training with the same volume as in the previous loading phase, whereas TAPER decreased their training volume by 50%. Endurance performance was assessed and muscle biopsies were taken before the start of the intervention period (t0), after 9 weeks of loading (t9) and after the two weeks of continued training (t11) or tapering (t9 + 2).

### 2.2 Participants

Twenty‐four trained German male cyclists were initially recruited for this study. Nine participants did not complete the intervention period due to prolonged illness or injury (*n* = 5), low training adherence (*n* = 3) or missing performance diagnostics (*n* = 1). Therefore, 15 participants were included in the physiological analysis (TAPER: *n* = 8; age: 26.0 ± 7.8 yrs.; height: 185.3 ± 6.0 cm; body mass: 79.7 ± 5.2 kg; VO_2_max: 59.3 ± 4.7 mL∙kg^−1^∙min^−1^; CON: *n* = 7; age: 32.7 ± 10.4 yrs.; height: 182.1 ± 7.2 cm; body mass: 77.6 ± 4.0 kg; VO_2_max: 59.8 ± 4.4 mL∙kg^−1^∙min^−1^). Prior to all testing, the medical history of all participants was assessed through a standardized questionnaire to ensure they were healthy and physically fit. Participants were informed about all possible risks and gave their written informed consent before participating in the study. This study was approved by the Ethics Committee of the German Sports University (37/2015) and conducted in accordance with the Declaration of Helsinki.

### 2.3 Training

**FIGURE 1:**
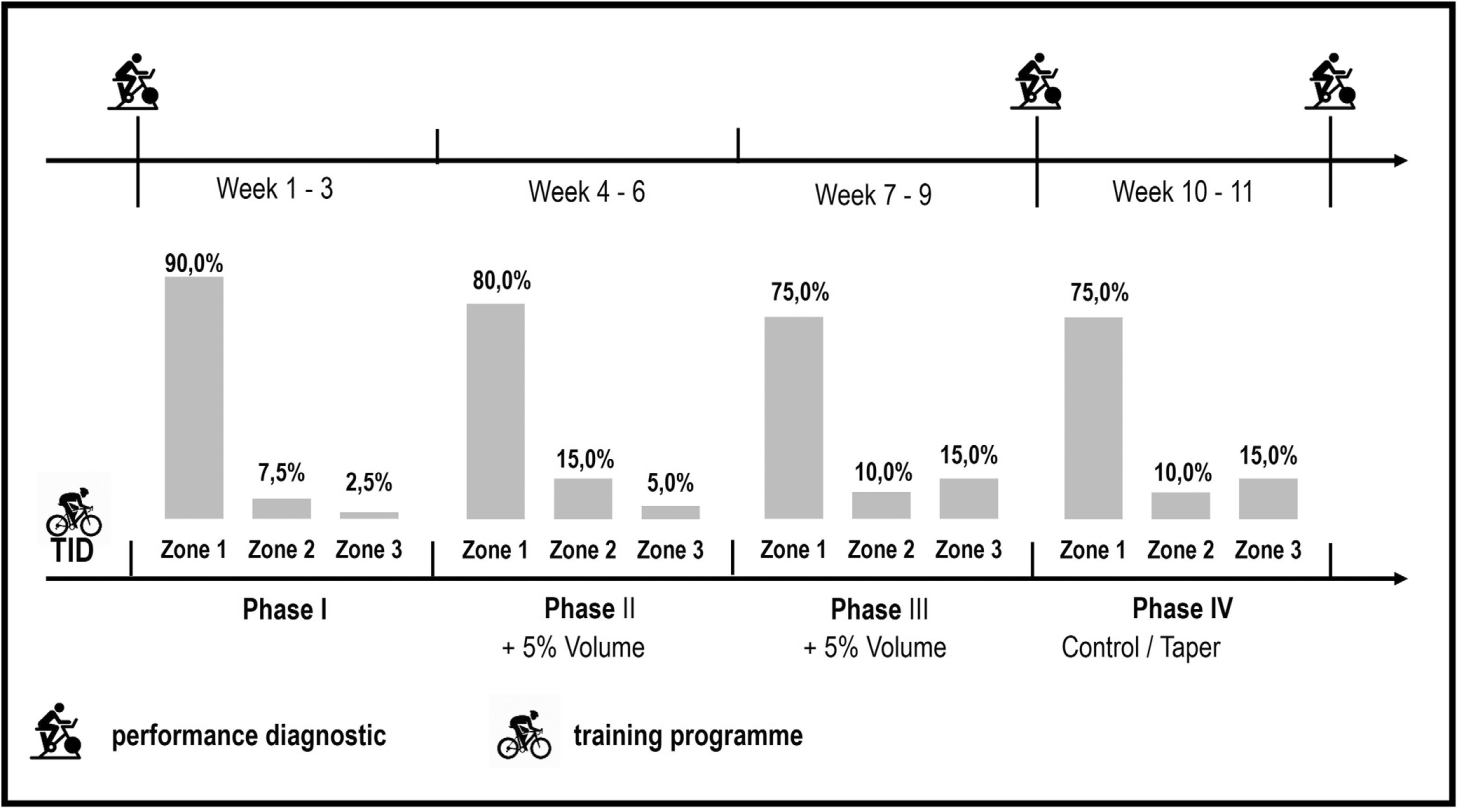
Training design of the 11‐week training intervention. Participants were separated into two groups after the 9‐week loading phase. TID, training intensity distribution. Zone 1, Training intensity below the first lactate threshold (LT_1_); Zone 2, Training intensity between LT_1_ and LT_2_; Zone 3, Training intensity above LT_2_.

The training was performed individually by each athlete following specific guidelines. The loading phase was split into three mesocycles of 3 weeks each. Initially, the training volume for each athlete was kept similar to the 3 weeks prior to inclusion in the study. Thereafter, training volume was increased by 5% every 3 weeks. The training intensity distribution (TID) followed a three training zone model (Seiler & Kjerland, 2006). Zone 1 was defined as training below the first lactate threshold (LT_1_), Zone 2 was defined as training between LT_1_ and the second lactate threshold (LT_2_) and Zone 3 was defined as training above LT_2_. Training intensity progressively increased throughout phase I–III (Figure 1). Subsequently, after 9 weeks of training, participants were matched into TAPER and CON based on their baseline V̇O_2_max, peak power output (PPO_R_) and training volume. During the last 2 weeks of training, the TID was maintained in both training groups. However, while CON kept the same training volume, TAPER reduced training volume by 50% in the last 2 weeks. The training intensity was maintained in both groups in order not to compromise maximal exercise performance.

Athletes collected power and heart rate data of each training session and reported this weekly. Athletes were instructed to use their own training monitors and calibrate these according to the manufacturer's instructions. To assure compliance and to ensure predefined TID was achieved, data were analyzed weekly and feedback was provided to the participants. To further quantify training load, the Lucias Training Impulse (luTRIMP) (Lucía et al., 2003) and the Training Stress Score as proposed by Coggan (Coggan, 2003) were calculated as measures of internal and external training load, respectively.

### 2.4 Performance testing

Athletes were instructed to refrain from any physical activity and alcohol 48 h prior to testing and to keep their nutritional intake before each testing session similar. All performance tests were performed on a SRM ergometer (Schoberer Rad Messtechnik SRM GmbH, Jülich, Deutschland) using the participants pedals, shoes, and saddle. The height/width of the saddle and handlebar were determined according to the participant's preferences in the first testing session and were kept constant for all further testing sessions. The time of day and the order of the testing procedures were kept the same for each testing session as follows: (1) submaximal incremental test, (2) sprint test, and (3) a maximal ramp test. Athletes were allowed to drink water ad libitum.

The submaximal incremental test started at 120 W and was increased by 30 W every 3 min. Capillary blood samples and ratings of perceived exertion (RPE; BORG Scale 6–20) were collected at the end of each increment. The test was terminated after the participant reached a blood lactate concentration of 4 mmol∙L^−1^. Afterwards, an active recovery (i.e., cycling below 150 W) was performed until blood lactate concentration decreased below 1.5 mmol∙L^−1^, followed by a maximal isokinetic 15‐s sprint test at a predefined cadence of 120 rpm (Wahl et al., 2017). Participants performed the sprint in a seated position and were verbally encouraged to give maximal performance. To determine maximal lactate accumulation rate (VLamax), capillary blood samples were collected every minute for 10 min after the sprint test. The subsequent ramp protocol commenced at 120 W and was increased every minute by 20 W. Participants were verbally encouraged to give maximal performance. Maximal criteria were set as voluntary exhaustion, cadence below 60, respiratory exchange ratio (RER) ≥1.1 and RPE ≥18.

Cardiorespiratory data were recorded using a stationary breath‐by‐breath gas analyzer (Metalyzer® 3B; Cortex Biophysik GmbH, Leipzig, Germany) and interpolated for values every second during both the incremental and ramp tests. The spirometer was calibrated weekly with a reference gas and before each test with ambient air according to the manufacturer's specifications. Heart rate (Polar H7 Sensor; Polar Electro, Kempele, Finland) was recorded at a sampling frequency of 1 Hz throughout the incremental and ramp tests. Power data was recorded at a sampling frequency of 1 Hz for the incremental and ramp tests and 10 Hz for the sprint test.

### 2.5 Performance parameters

V̇O_2_max was defined as the highest 30‐s moving average oxygen uptake. Peak power output (PPO)_R_ was defined as the highest 60‐s moving average power output during the ramp test. LT_1_ was defined as the first rise in the blood lactate curve and was determined by the point at which the slope of the curve reached 0.02 (Zwingmann et al., 2019). LT_2_ was determined at a blood lactate concentration of 4 mmol∙L^−1^ (Mader et al., 1976).

Peak power output of the sprint test (PPO_S_) was defined as the highest single maximal power output recorded. Mean power output of the sprint test (MPO_S_) was defined as the average power output from the beginning of the test until the first decrease in cadence. Additionally, VLamax was determined as described previously (Quittmann et al., 2021; Wahl et al., 2017).

### 2.6 Muscle biopsies

Biopsies were taken at t0, t9, and t9 + 2/t11 from the vastus lateralis muscle of the same leg of both the controls and the taper subgroups on the day (24 h before) before performance testing. Participants were instructed to refrain from any physical activity and alcohol 48 h prior to the biopsy. One hour before the biopsy, participants consumed a standardized meal in the form of an energy drink (Fresubin® protein energy DRINK, Fresenius Kabi Deutschland GmbHm Bad Homburg, Germany; 1260 kJ) and 0.5 L of water in order to equalize the caloric situation between participants and timepoints. For the biopsy, the incision site was anesthetized and a hollow needle was inserted into the muscle. The Bergstrom technique allows for approximately 200 mg of muscle tissue to be collected. A vacuum was used to remove a muscle sample (approx. 500 fibers). A part of the tissue was cooled in nitrogen‐cooled isopentane and stored at −80°C for fiber‐typing analysis, while the rest of the tissue was frozen directly in liquid nitrogen and stored at −80°C for proteome analysis.

### 2.7 Fiber type analysis

For immunohistochemical staining of type I fibers, 7 μm sections of muscle tissue were cut using a LEICA Cryostat (LEICA CM 1900®, Nussloch, Germany) and mounted on slides. Type I expressing fibers were stained with anti‐A4951 (1:200, Developmental Studies Hybridoma Bank, Iowa, USA, catalogue no A4951) and anti‐mouse (1:400, Dako, Hamburg, Gemany, catalogue no E0433) and alkaline phosphatase (1:400, Dako, Hamburg, Germany, catalogue no D0396).

Non‐staining fibers were considered Type II fibers. These stains were then used to determine the ratio of type I versus type II fibers.

### 2.8 Proteomic analysis

Proteins were extracted by homogenizing aliquots of muscle biopsies (about 20 mg) in 400 μL (0.05/1, w/v) of a cold buffer containing 20 mM Tris pH 7.5, 150 mM NaCl, 1 mM EDTA, 2.5 mM Na_2_H_2_P_2_O_7_, 1 mM β‐C_3_H_7_O_6_PNa_2_, 1 mM Na_3_VO_4_, 1 mM PMSF, 1% (v/v) Triton X100, plus a protease inhibitory cocktail (Sigma Aldrich, Milan, Italy, catalogue no P8340) The samples used for proteomic analysis were randomly selected, avoiding those evidencing hemolysis, capable of negatively affecting the subsequent proteomic workflow. The protein concentration of each homogenate sample was evaluated spectrophotometrically, using a commercial Protein assay colorimetric kit (Protein Assay Dye, Bio‐Rad Hercules, CA, USA, catalogue no Bio‐Rad Quick Start™ Bradford 1x Dye Reagent 5,000,205), as specified by the manufacturer. Protein extracts of each subject were used for quantitative proteomic analysis. Peptide preparations were carried out as previously reported (Mauceri et al., 2024), with trypsin as the proteolytic enzyme in an E/S ratio of 1/40 w/w. The resulting peptide mixtures from each protein sample were tagged with the TMTpro™ 16plex Label Reagent Set (Thermo Fisher Scientific, Waltham, MA, USA, catalogue no. Thermo Fisher Scientific TMTpro™ 16plex Label Reagent Set A44521), at 25°C, in agreement with the manufacturer's instructions. Using 50 μg of proteins per sample, two parallel sets of quantitative proteomic experiments were performed, each on muscle protein extracts from *N* = 5 participants (for 3 time points) from controls or tapered participants, thus employing 15 out of the 16 TMTpro reagents, for a total of *N* = 10 participants (for 3 time points). For each of the two proteomic experiments, the 16th channel of the TMTpro 16plex Label Reagent Set was used to tag a pool of all analyzed samples with the aim to bridge the two experimental sets and allow data normalization and comparison.

The analysis of TMT‐labeled peptide fractions was carried out in technical triplicate on a nanoLC‐ESI‐Q‐Orbitrap‐MS/MS platform (Thermo Fisher Scientific), as previously described (Mauceri et al., 2024). Global results, consisting of protein identification and relative protein quantification across all samples, were obtained by combining two separately carried out sets of nanoLC‐ESI‐Q‐Orbitrap‐MS/MS analyses by the Proteome Discoverer (PD) 2.4 software (Thermo Fisher Scientific), enabling the database search by the Mascot algorithm v. 2.4.2 (Matrix Science, London, UK), using the following criteria: *Homo sapiens* UniProtKB protein database (82,492 protein sequences, 10/2023) including the most common protein contaminants; TMTpro adduction at Lys and peptide N‐terminus, and carbamidomethylation at Cys as fixed modifications; oxidation at Met, deamidation of Asn and Gln, pyroglutamate formation at N‐terminal Gln, and phosphorylation at Ser, Thr, and Tyr as variable modifications. Peptide and fragment mass tolerance were set to ±10 ppm and ±0.02 Da, respectively. Proteolytic enzyme and maximum number of missed cleavages were set to trypsin and 2, respectively. All protein entries were considered correctly identified only when False Discovery Rate and *Q*‐value were both ≤0.01.

In particular, Differential Represented Proteins (DRPs) were identified setting a fold change (i.e., magnitude of protein quantity changes among samples) with cut‐off values set at ≥1.30/≤0.77. Subsequently, the *on‐line* available Metascape software (Zhou et al., 2019) (https://metascape.org/gp/index.html#/main/step1) was used to obtain meaningful functional information from the lists of identified DRPs, which firstly ran Gene Ontology enrichment analysis (for the categories “Cellular Component”, “Molecular Function”, and “Biological Process”), secondly highlighted protein–protein interaction (PPI) networks (using the databases STRING, BioGrid, OmniPath, and InWeb_IM9) and, finally, automatically extracted, through the Molecular Complex Detection algorithm (MCODE) (Bader & Hogue, 2003), protein complexes embedded in the previously identified PPI networks with the aim to infer more biologically interpretable results.

### 2.9 Statistical analysis

All physiological data are displayed as mean ± standard deviation (SD). Statistical analysis was performed using SPSS 27.0 (SPSS, IBM Statistics, New York, NY, USA). Residual histograms and Q‐Q plots were visually checked for homoscedasticity and normality, and a Shapiro–Wilk test was performed prior to significant analysis. Due to baseline differences in the performance data, data from t9 and t11 were normalized to baseline values by subtracting data from t0 for t9 and t11/t9 + 2, respectively (i.e., t9 – t0 and t11/t9 + 2 – t0). For time and interaction effects, a mixed factorial analysis of variance (ANOVA) was performed with Bonferroni correction for post‐hoc tests. Timepoints (i.e., t0, t9, and t11) were defined as within‐group variables, while TAPER and CON were set as the between‐group variables. Effect sizes for main effects of the ANOVA were reported as partial *η* ^2^. Between‐group differences of training phases were assessed by a one‐way ANOVA. Associations between training and performance were tested with the Pearson product–moment correlation coefficient *r* (95% confidence intervals). In analogy, for fiber‐type percentage ratio analysis, differences in the ratio of type I and II fibers were assessed by one‐way ANOVA with Bonferroni's multiple comparison's post‐hoc test using Graphpad Prism 8.0 (Graphpad, San Diego, CA, USA). Statistics tools within the Proteome Discoverer 2.4 environment were used to verify the normal distribution of proteomic data and to carry out one‐way ANOVA tests to calculate *p*‐values for quantitative ratios, with Tukey HSD as the post‐hoc test. For all tests, the significance level was fixed at a *p* ≤ 0.05. All comparisons were performed considering the variance of each participant selected for proteomic analysis.

## 3 RESULTS

### 3.1 Training

Weekly training volume and training intensity distribution (TID) per training phase are displayed in Tables 1 and 2. In addition, the mean weekly training stress score (TSS) and Lucas training impulse (lucTRIMP) can be found in Table S7. Training volumes did not differ significantly between groups for training phases I–III, but were significantly higher in controls (CON) in training phase IV compared to tapering (TAPER) (*p* = 0.001). A training volume reduction of 47.4 ± 6.1% was achieved in the tapering phase in TAPER. Furthermore, TID did not differ between groups (all *p* > 0.05).

**TABLE 1:** Weekly training volume per training phase for TAPER and CON groups. Training volume was the same for both groups and increased by 5% in phases I–III. In phase IV, the tapering reduced their training volume by 50%.

	TAPER (h)	CON (h)	Between‐group *p*
Training Phase I	8.1 ± 2.7	9.4 ± 1.7	0.258
Training Phase II	9.3 ± 3.8	11.8 ± 4.3	0.235
Training Phase III	10.4 ± 3.4	11.1 ± 2.4	0.645
Training Phase IV	4.8 ± 1.3	10.9 ± 3.6	0.001

**TABLE 2:** TID per training phase for TAPER and CON groups. Both groups followed the same training intensity distribution throughout the intervention period. Zone 1, training below first lactate threshold (LT_1_); Zone 2, training between LT_1_ and LT_2_; Zone 3, training above LT_2_.

	Aim (%)	TAPER (%)	CON (%)	Between‐group *p*
Training Phase I				
Zone 1	90	83.6 ± 9.5	84.4 ± 5.3	0.834
Zone 2	7.5	12.8 ± 6.1	11.5 ± 3.9	0.656
Zone 3	2.5	3.7 ± 3.8	4.1 ± 1.9	0.841
Training Phase II				
Zone 1	80	80.1 ± 4.5	77.4 ± 5.4	0.316
Zone 2	15	14.9 ± 2.7	16.7 ± 3.6	0.309
Zone 3	5	5.1 ± 3.3	5.7 ± 2.0	0.663
Training Phase III				
Zone 1	75	76.9 ± 4.4	73.9 ± 3.6	0.181
Zone 2	10	13.0 ± 4.1	13.7 ± 2.3	0.796
Zone 3	15	10.2 ± 2.5	12.4 ± 2.8	0.092
Training Phase IV				
Zone 1	75	73.5 ± 6.5	75.4 ± 2.8	0.484
Zone 2	10	14.4 ± 4.8	11.7 ± 3.4	0.243
Zone 3	15	12.1 ± 5.3	12.9 ± 1.5	0.709

### 3.2 Maximal aerobic and anaerobic performance

**FIGURE 2:**
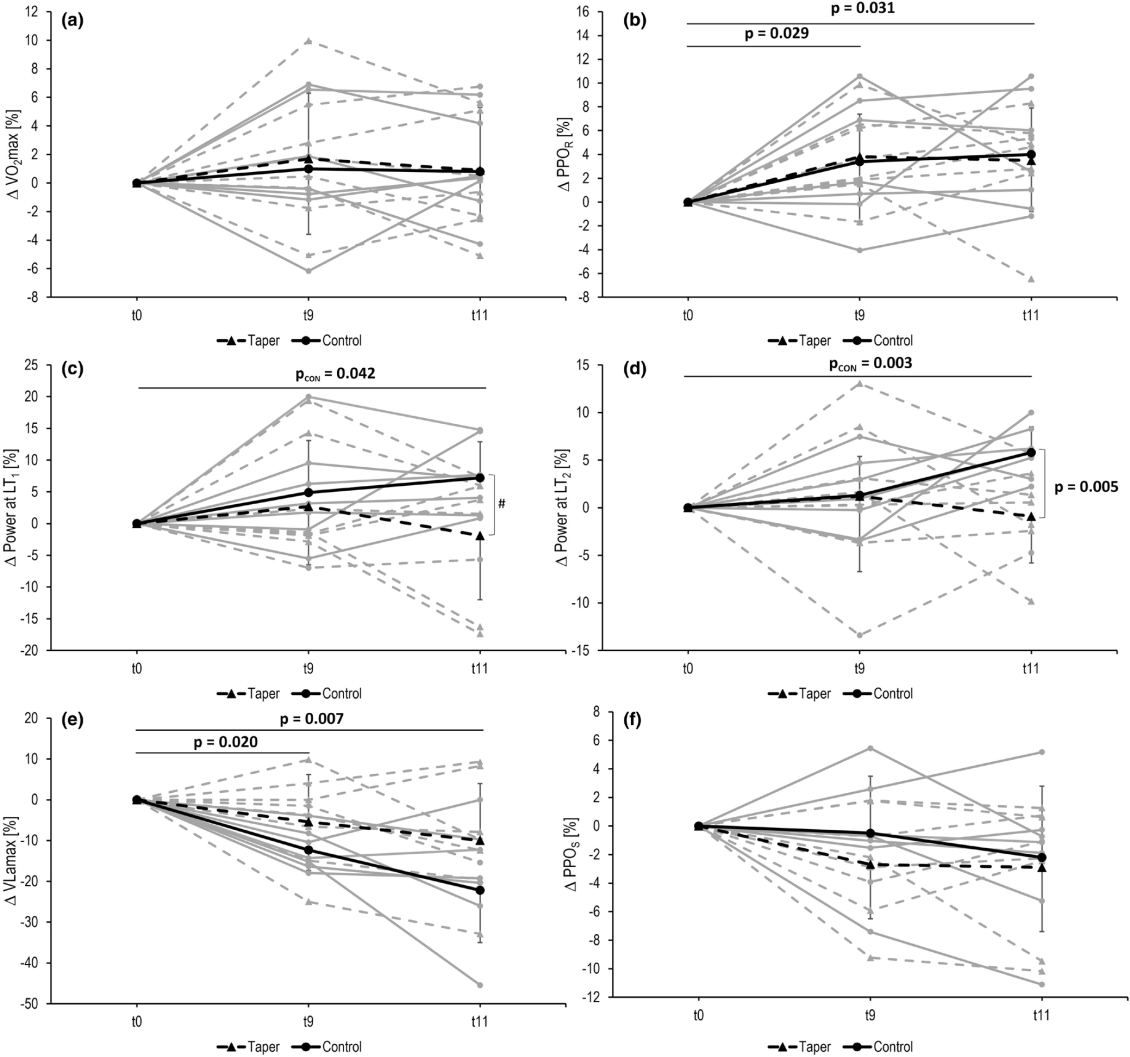
Change in relative VO_2_max (a), relative peak power output of the ramp test (PPO_R_, b), first lactate threshold (LT_1_, c), second lactate threshold (LT_2_, d), maximal lactate accumulation rate (Vlamax, e) and peak power output in the sprint test (PPO_S_, f) throughout the intervention period for the tapering and control group. NB: Groups were separated after 9 weeks of training (t9). Absolute *p*‐values of the differences between conditions are given in the figure.

Baseline values of all parameters measured related to aerobic and anaerobic performance are shown in Table 3 and Figure 2. For V̇O_2_max, no significant main effect was observed for time (*p* = 0.524, *η* ^2^ = 0.049) or interaction (*p* = 0.910, *η* ^2^ = 0.007) (Figure 2a). For PPO_R_, a significant main effect was found for time (*p* = 0.009, *η* ^2^ = 0.305) but not interaction (*p* = 0.931, *η* ^2^ = 0.005) (Figure 2b). PPO_R_ increased across both groups from t0 to t9 (+3.6 ± 4.3%, *p* = 0.029) as expected, and it was also increased from t0 to t11 (+3.7 ± 4.4%, *p* = 0.031).

**TABLE 3:** Baseline values (t0) of performance parameters for TAPER and CON groups.

	TAPER	CON	Between‐group *p*
VO_2_max (mL∙kg^−1^∙min^−1^)	59.3 ± 4.7	59.8 ± 4.4	0.831
PPO_R_ (W)	397 ± 36	394 ± 29	0.803
LT_1_ (W)	222 ± 37	223 ± 13	0.963
LT_2_ (W)	293 ± 42	302 ± 16	0.632
PPO_S_ (W)	1257 ± 186	1070 ± 164	0.061
MPO_S_ (W)	1038 ± 116	870 ± 108	0.012
VLamax (mmol∙l^−1^∙s^−1^)	0.7 ± 0.1	0.5 ± 0.2	0.033

Abbreviations: LT_1_, first lactate threshold; LT_2_, second lactate threshold; MPO_S_, mean power output in the sprint test; PPO_R_, relative peak power output of the ramp test; PPO_S_, peak power output in the sprint test; VLamax, maximal lactate accumulation rate;VO_2_max, maximal oxygen uptake.

For LT_1_, no significant main effect was found for time (*p* = 0.304, *η* ^2^ = 0.087) or interaction (*p* = 0.082, *η* ^2^ = 0.175). However, at t11 power at LT_1_ was significantly higher in CON compared to TAPER (238 ± 11 W vs. 216 ± 28 W, *p* = 0.05). Furthermore, in CON power at LT_1_ increased from t0 to t11 (+7.2 ± 5.7%, *p* = 0.042), while there was no significant change in the TAPER (*p* > 0.05) (Figure 2c).

For LT_2_, no significant main effect was found for time (*p* = 0.114, *η* ^2^ = 0.303) but there was a significant main effect for interaction (*p* = 0.016, *η* ^2^ = 0.042). At t11, power at LT_2_ was significantly higher in CON compared to TAPER (319 ± 13 W vs. 290 ± 42 W, *p* = 0.005). Furthermore, in CON, power at LT_2_ increased from t0 to t11 (+5.8 ± 2.7%, *p* = 0.003), while there was no significant change in TAPER (*p* > 0.05) (Figure 2d).

For VLamax, a significant main effect was observed for time (*p* < 0.001, *η* ^2^ = 0.462) but not for interaction (*p* = 0.749, *η* ^2^ = 0.022). VLamax decreased across both groups from t0 to t9 (−8.3 ± 9.3%, *p* = 0.02) as expected, and was also decreased from t0 to t11 (−14.2 ± 14.4%, *p* = 0.007) (Figure 2e). Analysis of PPO_S_ and MPO_S_ showed no significant main effect for time (PPO_S_: *p* = 0.682, *η* ^2^ = 0.029, MPO_S_: *p* = 0.918, *η* ^2^ = 0.007) or for interaction (PPO_S_: *p* = 0.456, *η* ^2^ = 0.059, MPO_S_: *p* = 0.103, *η* ^2^ = 0.161) (Figure 2f).

### 3.3 Differential proteomic analysis in vastus lateralis muscle

Time‐point t0, t9, t9 + 2, and t11 muscle biopsy material of a selection of 5 participants per group was used for quantitative proteomic analysis. Quantitative protein determination was performed using a Tandem Mass Tag (TMT)‐based proteomic approach (Mauceri et al., 2024) followed by bioinformatic data elaboration. This allowed the identification of a total of 2179 proteins in muscle samples and relative quantification of 2162 unique protein entries (Table S1) using PD 2.4 software, which performed the analysis of proteomic data of all participants independently. Relying on precise and accurate quantitation features of the TMT‐based proteomic approach, proteins with a concomitant fold change value ≥1.3 or ≤0.77 and an abundance ratio *p*‐value ≤0.05 were considered as differentially represented. Although all conditions were compared simultaneously through the PD software, for clarity, we describe the following binary comparisons: (a) t0 versus t11; (b) t9 versus t11; (c) t0 versus t9 + 2; (d) t9 versus t9 + 2; (e) t9 + 2 versus t11.

**FIGURE 3:**
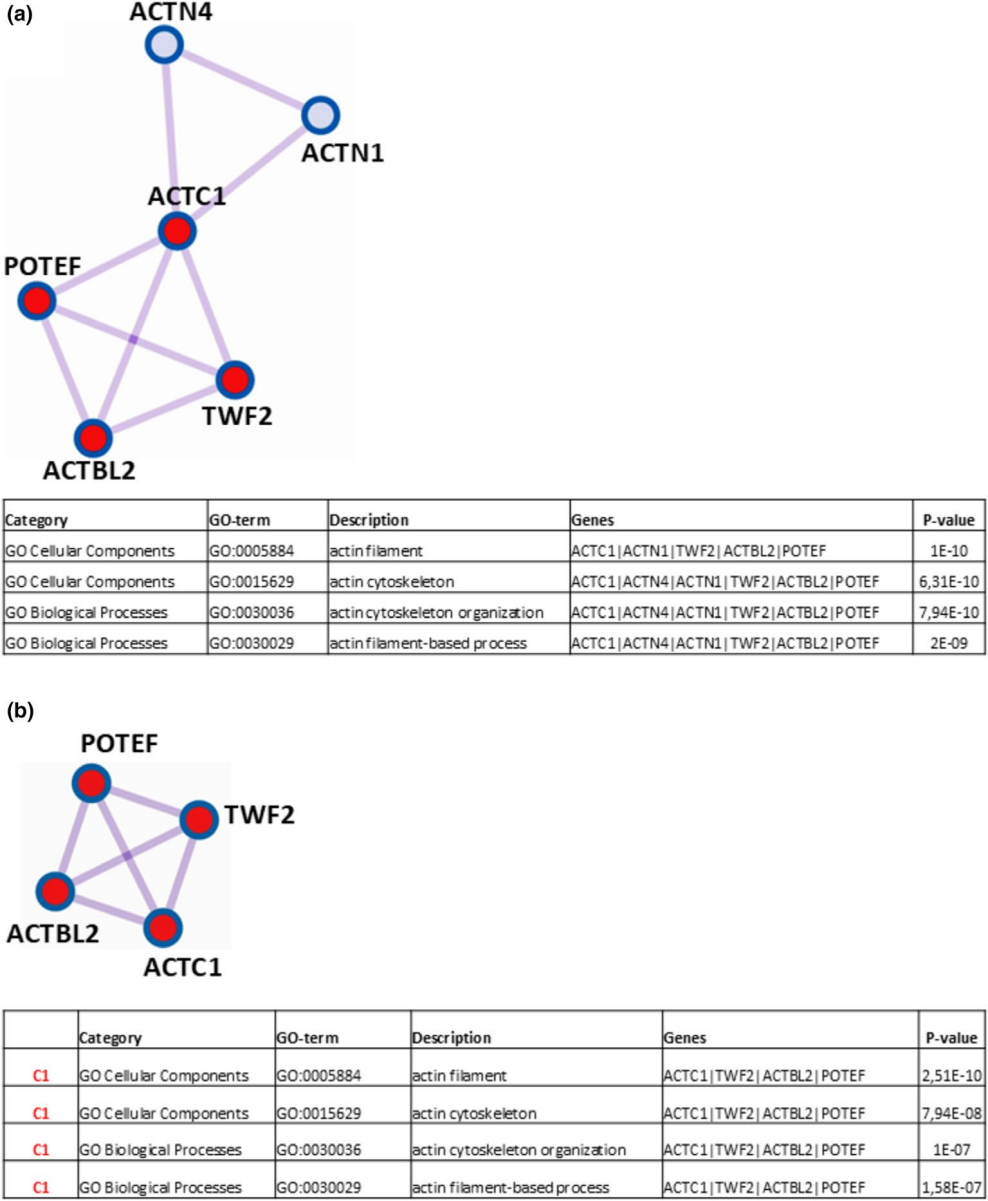
Protein–protein interaction (PPI) network for the over‐represented proteins at t11 in the comparison “t0 vs. t11” resulting from Mestascape analysis and subsequent pathway and process enrichment (GO‐enrichment) analysis of the corresponding proteomic results* (a). Protein cluster (C1) identified for the over‐represented proteins at t11 by Molecular Complex Detection algorithm and relative GO‐enrichment analysis of results* (b). *Shown in the following table are the four most significantly enriched Gene Ontology terms (including GO‐Category, GO‐term identifier, description, involved genes, and *p*‐value).

For the binary comparison “t0 vs. t11” a total of 13 proteins showed a differential representation, of which 11 proved to be significantly over‐represented and 2 down‐represented at t11. To attribute a biological meaning to Differential Represented Proteins (DRPs), the on‐line accessible Metascape software (Quittmann et al., 2021) was used to perform functional enrichment analysis. In particular, this software was able: (i) to carry out Gene Ontology enrichment (focused on the categories “Cellular Component”, “Molecular Function”, and “Biological Process”) from the lists of identified DRPs; (ii) to highlight interactome (PPI) networks for subsets of DRPs forming physical interactions with other members in each list, which could help illuminate biochemical complexes/components governing biological manifestations; (iii) to shed light on protein complexes within the previously identified PPI networks, operating the Molecular Complex Detection algorithm (MCODE) (Mauceri et al., 2024), with the aim of gaining a deeper comprehension of the biological significance of proteomic data. Applying this sequential pipeline to the 11 over‐represented proteins at t11 from the comparison “t0 vs. t11”, Metascape returned the PPI network and the Protein Complex (C1) displayed in Figure 3a,b, respectively, each with a table indicating the four most significantly enriched Gene Ontology terms (GO‐term), where, for brevity, proteins are designated by the corresponding gene names.

Proteins belonging to the detected interactome network (6 species) and to C1 cluster (4 species) are reported in Table 4, together with the corresponding UniProtKB accessions, protein names, protein abundances (Ab_t0_ and Ab_t11_), fold changes (11/0), and ratio *p*‐values, as calculated by PD software.

**TABLE 4:** Interacting over‐represented proteins at t11, as determined by Metascape analysis of results from the comparison “t0 vs. t11”. Reported are UniProtKB accessions, proteins and corresponding gene names, protein abundances at t0 (Ab_t0_) and t11 (Ab_t11_), abundance ratios Ab_t11_/Ab_t0_ (11/0) and corresponding ratio *p*‐values. Proteins belonging to the cluster (C1) detected by MCODE algorithm (Figure 3b) are also indicated.

UniProtKB Acc.	Protein	Gene	Ab_t0_	Ab_t11_	11/0	*p*‐Value	MCODE
P68032	Actin, alpha cardiac muscle 1	ACTC1	72.1	226.7	3.14	0.0135	C1
A5A3E0	POTE ankyrin domain family member F	POTEF	77.0	171.7	2.23	0.0097	C1
Q562R1	Beta‐Actin‐like protein 2	ACTBL2	93.6	151.5	1.62	0.0213	C1
A0A7I2V2U8	Twinfilin Actin binding protein 2	TWF2	92.4	130.2	1.41	0.0313	C1
H7C144	Alpha‐actinin‐4	ACTN4	89.2	123.1	1.38	0.0067	
A0A7I2V4Y4	Alpha‐actinin‐1	ACTN1	90.1	120.1	1.33	0.0184	

In detail, pathway and process enrichment analysis applied to the obtained PPI network (Figure 3a) revealed the GO‐terms “actin filament”, “actin cytoskeleton”, “actin cytoskeleton organization”, and “actin filament‐based process” as the four (out of a total of thirty‐two) statistically most significant increased (*p* « 0.01), which arose from the protein species actin, alpha cardiac muscle 1 (ACTC1), alpha‐actinin‐4 (ACTN4), alpha‐actinin‐1 (ACTN1), twinfilin actin binding protein 2 (TWF2), beta‐actin‐like protein 2 (ACTBL2), and POTE ankyrin domain family member F (POTEF). To extract more information from the data, Metascape software was able to detect by the MCODE algorithm, within the PPI network, the proteins ACTC1, TWF2, ACTBL2, and POTEF as densely connected in the cluster C1 (Figure 3b), for which pathway and process enrichment analysis reconfirmed as the four (out of a total of nine) statistically most significant enhanced (*p* « 0.01) GO‐terms “actin filament”, “actin cytoskeleton”, “actin cytoskeleton organization”, “actin filament‐based process”. Altogether, these findings indicated that the observed effects related to actin/actin organization may be linked to a gain in muscle strength at t11 with respect to the t0 condition. However, since muscle strength was not assessed in this study, these assumptions cannot be physiologically confirmed. All data not included here for the comparison “t0 vs. t11”, regarding DRPs identification and functional enrichment/PPI network/MCODE analyses, are reported in Table S2.

The binary comparison “t9 vs. t11” showed a total of 10 DRPs, each being significantly over‐represented at t11. In this case, Metascape software was able to highlight an interactome network constituted by actin, alpha cardiac muscle 1 (ACTC1), POTE ankyrin domain family member F (POTEF), beta‐actin‐like protein 2 (ACTBL2), actinin alpha 4 (ACTN4), and ataxin 2 (ATXN2) (Table 5). For these species, pathway and process enrichment analysis recognized “actin cytoskeleton”, “actin cytoskeleton organization”, “actin filament”, and “actin filament‐based process” as the four (out of a total of six) statistically most relevant increased GO‐terms (Table 6). On the other hand, the MCODE algorithm could not identify peculiar molecular complexes within this PPI network. Although a clear protein cluster, as for the parallel comparison “t0 vs. t11”, was missing, current observations emphasized that the recorded enhancement effects were again related to actin/actin organization and, accordingly, to a probable gain in muscle strength, even if no physiological confirmation can be provided in this study. Considering that no functional enrichment was observed for the comparison “t0 vs. t9” (data not shown), this likely increase in muscle strength could be assumed as mainly achieved during the last 2 training weeks, paralleling the current binary comparison (“t9 vs. t11”) with the previous (“t0 vs. t11”). All information not included here for the comparison “t9 vs. t11”, regarding DRPs identification and functional enrichment/PPI network analyses, is reported in Table S3.

**TABLE 5:** Interacting over‐represented proteins at t11, as determined by Metascape analysis of results from the comparison “t9 vs. t11”. Reported are UniProtKB accessions, proteins and corresponding gene names, protein abundances at t9 (Ab_t9_) and t11 (Ab_t11_), abundance ratios Ab_t11_/Ab_t9_ (11/9), and corresponding ratio *p*‐values.

UniProtKB Acc.	Protein	Gene	Ab_t9_	Ab_t11_	11/9	*p*‐Value
P68032	Actin, alpha cardiac muscle 1	ACTC1	65.7	226.7	3.45	0.0085
A5A3E0	POTE ankyrin domain family member F	POTEF	91.6	171.7	1.88	0.0500
Q562R1	Beta‐Actin‐like protein 2	ACTBL2	89.6	151.5	1.69	0.0254
H7C144	Actinin alpha 4	ACTN4	94.5	123.1	1.30	0.0251
A0A5F9ZHW5	Ataxin 2	ATXN2	90.6	117.8	1.30	0.0500

**TABLE 6:** Pathway and process enrichment (GO‐enrichment) analysis applied to the PPI network identified for the over‐represented proteins at t11 in the comparison “t9 vs. t11”. Reported are the four most significantly enriched Gene Ontology terms (including GO‐Category, GO‐term identifier, description, involved genes, and *p*‐value).

Category	GO‐term	Description	Genes	*p*‐Value
GO Cellular Components	GO:0015629	Actin cytoskeleton	ACTC1|ACTN4|ACTBL2|POTEF	3.98E‐07
GO Biological Processes	GO:0030036	Actin cytoskeleton organization	ACTC1|ACTN4|ACTBL2|POTEF	5.01E‐07
GO Cellular Components	GO:0005884	Actin filament	ACTC1|ACTBL2|POTEF	6.31E‐07
GO Biological Processes	GO:0030029	Actin filament‐based process	ACTC1|ACTN4|ACTBL2|POTEF	7.94E‐07

**FIGURE 4:**
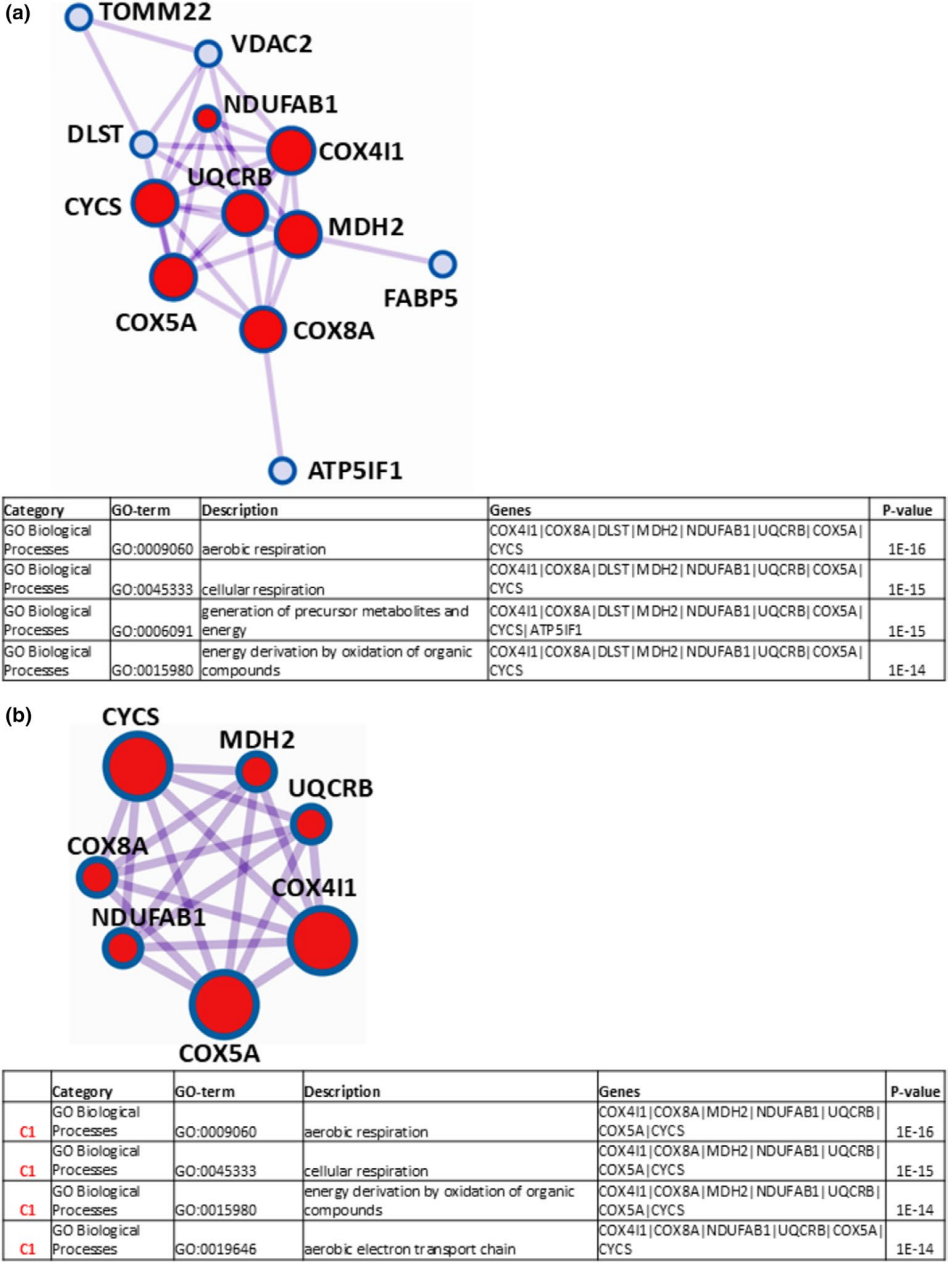
Protein–protein interaction (PPI) network for the over‐represented proteins at t9 + 2 in the comparison “t0 vs. t9 + 2” resulting from Mestascape analysis and subsequent pathway and process enrichment (GO‐enrichment) analysis of the corresponding proteomic results* (a). Protein cluster (C1) identified for the over‐represented proteins at t9 + 2 by Molecular Complex Detection algorithm and relative GO‐enrichment analysis of results* (b). *Shown in the following table are the four most significantly enriched Gene Ontology terms (including GO‐Category, GO‐term identifier, description, involved genes, and *p*‐value).

The binary comparison “t0 vs. t9 + 2” showed a total of 25 DRPs, of which 13 were over‐represented and 12 down‐represented at t9 + 2. Regarding the 13 over‐represented proteins at t9 + 2, Metascape software indicated the PPI network and the protein complex (C1) displayed in Figure 4a,b, respectively, both with a table reporting the four most significantly enriched Gene Ontology terms, where proteins are indicated by the corresponding gene names.

Of the twelve protein species belonging to the identified PPI network and the seven of the C1 cluster are indicated in Table 7, together with the corresponding UniProtKB accessions, protein names, protein abundances (Ab_t0_ and Ab_t9+2_), fold changes (9 + 2/0), and ratio *p*‐values, which are calculated by PD software.

**TABLE 7:** Interacting over‐represented proteins at t9 + 2, as determined by Metascape analysis of results from the comparison “t0 vs. t9 + 2”. Reported are UniProtKB accessions, proteins and corresponding gene names, protein abundances at t0 (Ab_t0_) and t9 + 2 (Ab_t9+2_), abundance ratios Ab_t9+2_/Ab_t0_ (9 + 2/0), and corresponding ratio *p*‐values.

UniProt Acc.	Protein	Gene	Ab_t0_	Ab_t9+2_	9 + 2/0	*p*‐Value	MCODE
P13073	Cytochrome c oxidase subunit 4 isoform 1, mitochondrial	COX4I1	92.0	130.8	1.42	0.0121	C1
E5RHG9	Cytochrome b‐c1 complex subunit 7	UQCRB	86.7	117.6	1.36	0.0143	C1
P20674	Cytochrome c oxidase subunit 5A, mitochondrial	COX5A	93.4	126.7	1.36	0.0465	C1
P10176	Cytochrome c oxidase subunit 8A, mitochondrial	COX8A	92.4	122.3	1.32	0.0324	C1
C9JFR7	Cytochrome c	CYCS	95.3	124.7	1.31	0.0249	C1
P40926	Malate dehydrogenase, mitochondrial	MDH2	92.3	119.7	1.30	0.0089	C1
O14561	Acyl carrier protein, mitochondrial	NDUFAB1	95.4	125.0	1.31	0.0276	C1
Q9NS69	Mitochondrial import receptor subunit TOM22 homolog	TOMM22	89.4	119.1	1.33	0.0256	
Q9UII2	ATPase inhibitor, mitochondrial	ATP5IF1	96.5	128.2	1.33	0.0331	
Q01469	Fatty acid‐binding protein 5	FABP5	91.2	120.5	1.32	0.0129	
P36957	Dihydrolipoyllysine‐residue succinyltransferase component of 2‐oxoglutarate dehydrogenase complex, mitochondrial	DLST	93.2	122.2	1.31	0.0195	
P45880	Voltage‐dependent anion‐selective channel protein 2	VDAC2	89.9	117.6	1.31	0.0007	

When applying pathway and process enrichment analysis to the obtained PPI network (Figure 4a), the GO‐terms “aerobic respiration”, “cellular respiration”, “generation of precursor metabolites and energy”, and “energy derivation by oxidation of organic compounds” resulted as the four (out of a total of fifty‐one) statistically most relevant increased (*p* « 0.01), which derived from the proteins cytochrome c oxidase subunit 4 isoform 1, mitochondrial (COX4I1), cytochrome c oxidase subunit 8A, mitochondrial (COX8A), dihydrolipoyllysine‐residue succinyltransferase component of 2‐oxoglutarate dehydrogenase complex, mitochondrial (DLST), malate dehydrogenase, mitochondrial (MDH2), acyl carrier protein, mitochondrial (NDUFAB1), cytochrome b‐c1 complex subunit 7 (UQCRB), cytochrome c oxidase subunit 5A, mitochondrial (COX5A), and cytochrome c (CYCS). Then, Metascape software, operating the MCODE algorithm, recognized, within the protein network, the species COX4I1, COX8A, MDH2, NDUFAB1, UQCRB, COX5A, and CYCS as strongly connected in the cluster C1 (Figure 4b). For the latter, pathway and process enrichment analysis indicated “aerobic respiration”, “cellular respiration”, “energy derivation by oxidation of organic compounds”, and “aerobic electron transport chain” as the four (out of a total of forty) statistically most significant increased (*p* « 0.01) GO‐terms, thereby reconfirming previous observations. Therefore, these data showed that, with respect to t0, the t9 + 2 condition was characterized by a substantial shift of the muscle proteome towards structural/functional changes associated with an increased oxygen consumption. All data here not considered for the current binary comparison, regarding DRPs identification and functional enrichment/PPI network/MCODE analyses, are reported in Table S4.

Concerning down‐represented proteins at t9 + 2, Metascape functional enrichment analysis gave very poor results, revealing only the GO‐terms “calmodulin binding” (GO:0005516), and “oxidoreductase activity” (GO:0016491) as the statistically most significant decreased, while it did not unveil either a clear protein–protein interaction network or a protein cluster (Table S4).

**FIGURE 5:**
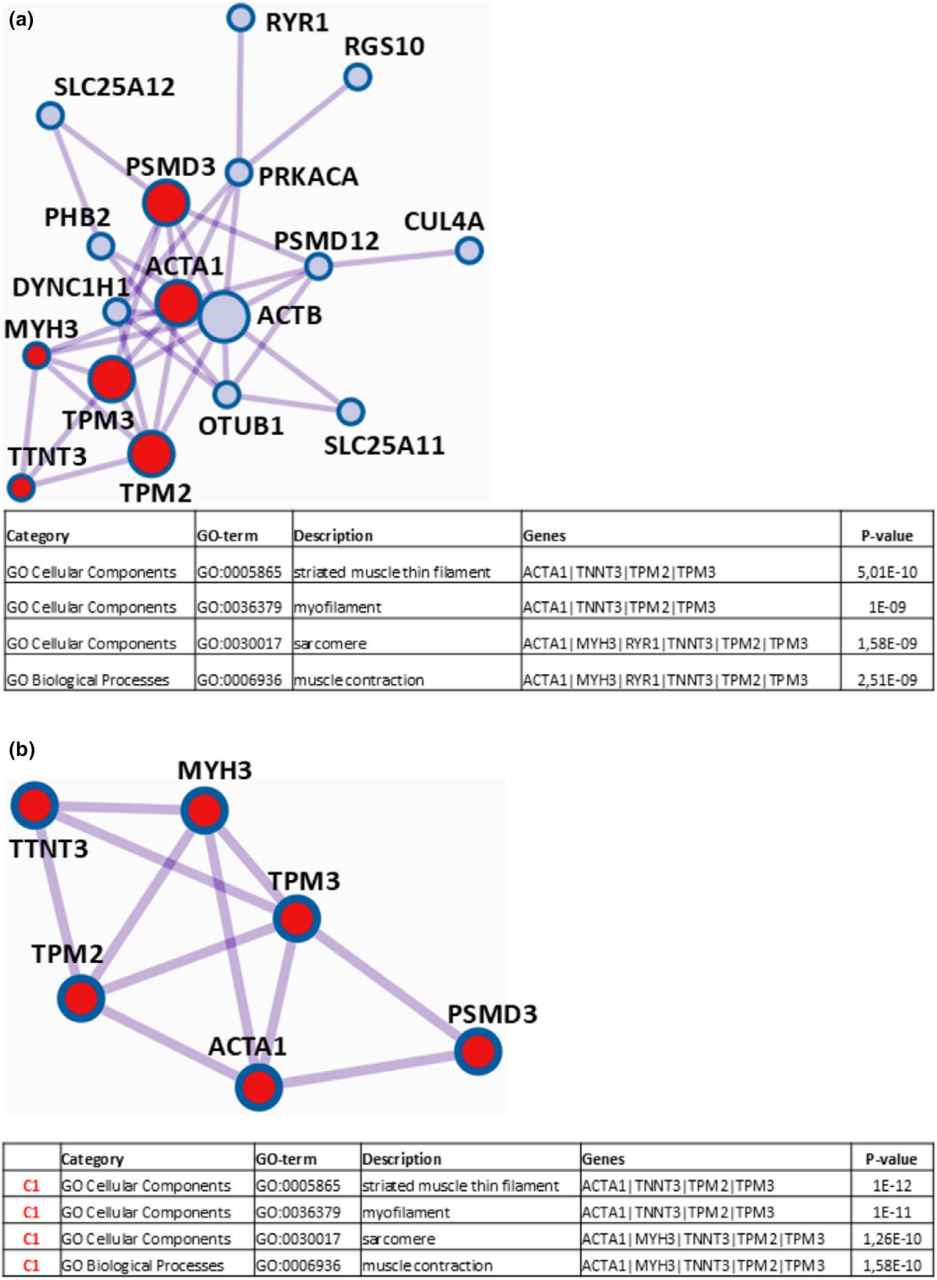
Protein–protein interaction (PPI) network for the down‐represented proteins at t9 + 2 in the comparison “t9 vs. t9 + 2” resulting from Mestascape analysis and subsequent pathway and process enrichment (GO‐enrichment) analysis of the corresponding proteomic results* (a). Protein cluster (C1) identified for the down‐represented proteins at t9 + 2 by Molecular Complex Detection algorithm and relative GO‐enrichment analysis of results* (b). *Shown in the following table are the four most significantly enriched Gene Ontology terms (including GO‐Category, GO‐term identifier, description, involved genes, and *p*‐value).

The binary comparison “t9 vs. t9 + 2” showed a total of 28 DRPs, of which 7 resulted over‐represented and 21 down‐represented at t9 + 2. Regarding the 21 down‐represented proteins at t9 + 2, Metascape analysis revealed the PPI network and the protein complex (C1) displayed in Figure 5a,b, respectively, each with a table indicating the four most significantly enriched Gene Ontology terms, where proteins are reported by the corresponding gene names.

Proteins belonging to the detected interactome network (17 species) and to the C1 cluster (6 species) are reported in Table 8, together with the corresponding UniProtKB accessions, protein names, protein abundances (Ab_t9_ and Ab_t9+2_), fold changes (9 + 2/9), and ratio *p*‐values, as calculated by PD software.

**TABLE 8:** Interacting down‐represented proteins at t9 + 2, as determined by Metascape analysis of results from the comparison “t9 vs. t9 + 2”. Reported are UniProtKB accessions, proteins and corresponding gene names, protein abundances at t9 (Ab_t9_) and t9 + 2 (Ab_t9+2_), abundance ratios Ab_t9+2_/Ab_t9_ (9 + 2/9) and corresponding ratio *p*‐values. Proteins belonging to the cluster (C1) detected by MCODE algorithm (Figure 5b) are also indicated.

UniProtKB Acc.	Protein	Gene	Ab_t9_	Ab_t9+2_	9 + 2/9	*p*‐Value	MCODE
P45378	Troponin T, fast skeletal muscle	TNNT3	114.4	88.2	0.77	0.0094	C1
P68133	Actin, alpha skeletal muscle	ACTA1	117.0	90.0	0.77	0.0055	C1
P06753	Tropomyosin alpha‐3 chain	TPM3	120.5	91.7	0.76	0.0456	C1
Q5TCU3	Tropomyosin beta chain	TPM2	119.9	90.4	0.75	0.0292	C1
O43242	26S proteasome non‐ATPase regulatory subunit 3	PSMD3	107.8	79.8	0.74	0.0378	C1
P11055	Myosin‐3	MYH3	115.5	80.9	0.70	0.0255	C1
A0A2R8Y793	Actin, cytoplasmic 1	ACTB	109.3	84.6	0.77	0.0426	
P17612	cAMP‐dependent protein kinase catalytic subunit alpha	PRKACA	111.1	85.7	0.77	0.0047	
Q02978	Mitochondrial 2‐oxoglutarate/malate carrier protein	SLC25A11	111.6	86.0	0.77	0.0433	
F5GYJ8	Ubiquitinyl hydrolase 1	OTUB1	106.5	82.3	0.77	0.0341	
O75746	Electrogenic aspartate/glutamate antiporter SLC25A12, mitochondrial	SLC25A12	108.9	83.2	0.76	0.0468	
A0A7P0T9C4	Dynein heavy chain, cytosolic	DYNC1H1	107.4	82.0	0.76	0.0200	
P21817	Ryanodine receptor 1	RYR1	108.8	82.3	0.76	0.0316	
O43665	Regulator of G‐protein signaling 10	RGS10	89.5	67.3	0.75	0.0008	
Q99623	Prohibitin‐2	PHB2	109.3	81.7	0.75	0.0463	
Q13619	Cullin‐4A	CUL4A	107.6	77.4	0.72	0.0477	
O00232	26S proteasome non‐ATPase regulatory subunit 12	PSMD12	110.2	75.8	0.69	0.0298	

Regarding the down‐represented proteins at t9 + 2, pathway and process enrichment analysis applied to the obtained PPI network (Figure 5a) recognized the GO‐terms “striated muscle thin filament”, “myofilament”, “sarcomere”, and “muscle contraction” as the four (out of a total of seventy‐three) statistically most relevant decreased (*p* « 0.01), which resulted from the protein species actin, alpha skeletal muscle (ACTA1), myosin‐3 (MYH3), ryanodine receptor 1 (RYR1), troponin T, fast skeletal muscle (TNNT3), tropomyosin beta chain (TPM2), and tropomyosin alpha‐3 chain (TPM3). With the aim to extract more information from the data, Metascape software could identify by the MCODE algorithm, among the species within the network, the proteins ACTA1, MYH3, TNNT3, TPM2, TPM3, and 26S proteasome non‐ATPase regulatory subunit 3 (PSMD3) as strongly interacting in the cluster C1 (Figure 5b). For the latter, pathway and process enrichment analysis highlighted the GO‐terms “striated muscle thin filament”, “myofilament”, “sarcomere”, and “muscle contraction” as the four (out of a total of twenty‐six) statistically most significant diminished (*p* « 0.01), thereby reinforcing previous findings. Accordingly, this outcome suggested that the observed effects related to muscle cell components/muscle contraction may be associated to a loss of muscle strength at t9 + 2 with respect to the condition prior to the tapering phase (t9). However, these assumptions cannot be confirmed from a physiological point of view, since muscle strength was not evaluated in this study. All information here not present for the comparison “t9 vs. t9 + 2”, regarding DRPs identification and functional enrichment/PPI network/MCODE analyses, is reported in Table S5.

Concerning the over‐represented proteins at t9 + 2, Metascape functional enrichment analysis provided not very informative results, with only the GO‐terms “carboxylic acid binding” (GO:0031406), “negative regulation of cell population proliferation” (GO:0008285), and “secretory granule lumen” (GO:0034774) as the statistically most relevant increased (*p* « 0.01), while PPI network/MCODE analyses could not generate any results evidencing PPI network/protein cluster(s) (Table S5).

For the binary comparison “t9 + 2 vs. t11” a total of 213 DRPs were identified, of which 42 resulted over‐represented and 171 down‐represented at t9 + 2. Regarding the over‐represented proteins at t9 + 2, Metascape software could detect an interactome network constituted by the 36 protein species reported in Table 9.

**TABLE 9:** Interacting over‐represented proteins at t9 + 2, as determined by Metascape analysis of results from the comparison “t9 + 2 vs. t11”. Reported are UniProtKB accessions, proteins and corresponding gene names, protein abundances at t9 + 2 (Ab_t9+2_) and t11 (Ab_t11_), abundance ratios Ab_t9+2_/Ab_t11_ (9 + 2/11) and corresponding ratio *p*‐values. Proteins belonging to the clusters (C1 and C2) detected by MCODE algorithm (Figure 6a,b) are also indicated.

UniProtKB Acc.	Protein	Gene	Ab_t9+2_	Ab_t11_	9 + 2/11	*p*‐Value	MCODE
B8ZWD1	Acyl‐CoA‐binding protein	DBI	124.4	77.8	1.60	0.0238	C1
C9JFR7	Cytochrome c	CYCS	124.7	78.9	1.58	0.0019	C1
O14561	Acyl carrier protein, mitochondrial	NDUFAB1	125.0	80.4	1.55	0.0026	C1
P20674	Cytochrome c oxidase subunit 5A, mitochondrial	COX5A	126.7	83.5	1.52	0.0068	C1
P10176	Cytochrome c oxidase subunit 8A, mitochondrial	COX8A	122.3	83.3	1.47	0.0036	C1
P14927	Cytochrome b‐c1 complex subunit 7	UQCRB	118	81.3	1.45	0.0132	C1
Q01469	Fatty acid‐binding protein 5	FABP5	120.5	88.6	1.36	0.0079	C1
P30405	Peptidyl‐prolyl cis‐trans isomerase F, mitochondrial	PPIF	116.1	86.2	1.35	0.0134	C1
P10606	Cytochrome c oxidase subunit 5B, mitochondrial	COX5B	117.3	90.4	1.30	0.0347	C1
P00441	Superoxide dismutase [Cu‐Zn]	SOD1	111.3	86.1	1.30	0.0032	C1
P30086	Phosphatidylethanolamine‐binding protein 1	PEBP1	114.4	88.5	1.30	0.0088	C1
P13073	Cytochrome c oxidase subunit 4 isoform 1, mitochondrial	COX4I1	130.8	76.4	1.71	0.0014	C2
O75947	ATP synthase subunit d, mitochondrial	ATP5PD	115.5	80.3	1.44	0.0457	C2
P40926	Malate dehydrogenase, mitochondrial	MDH2	119.7	87.5	1.37	0.0038	C2
P18859	ATP synthase‐coupling factor 6, mitochondrial	ATP5PF	114.6	85.5	1.34	0.0113	C2
Q5T7C4	High mobility group protein B1	HMGB1	126.7	77.5	1.63	0.0006	
Q9UII2	ATPase inhibitor, mitochondrial	ATP5IF1	128.2	83.2	1.54	0.0057	
F8W7Q4	Protein FAM162A	FAM162A	125.9	82.2	1.53	0.0239	
A0A0B4J2D5	Putative glutamine amidotransferase‐like class 1 domain‐containing protein 3B, mitochondrial	GATD3	118.6	81.4	1.46	0.0097	
Q32Q12	Nucleoside diphosphate kinase	NME1‐NME2	120.1	83.6	1.44	0.0044	
P36957	Dihydrolipoyllysine‐residue succinyltransferase component of 2‐oxoglutarate dehydrogenase complex, mitochondrial	DLST	122.2	86.5	1.41	0.0064	
O43583	Density‐regulated protein	DENR	118.2	84.6	1.40	0.0088	
P52566	Rho GDP‐dissociation inhibitor 2	ARHGDIB	117.5	84.1	1.40	0.0088	
A0A804HLK8	Leucine rich pentatricopeptide repeat containing	LRPPRC	120.9	86.7	1.39	0.0183	
Q16836	Hydroxyacyl‐coenzyme A dehydrogenase, mitochondrial	HADH	117.6	87.0	1.35	0.0048	
P09211	Glutathione S‐transferase P	GSTP1	116.7	86.8	1.34	0.0245	
P49773	Adenosine 5′‐monophosphoramidase HINT1	HINT1	112.2	84.3	1.33	0.0087	
O43676	NADH dehydrogenase [ubiquinone] 1 beta subcomplex subunit 3	NDUFB3	114.7	86.4	1.33	0.0383	
Q69YU5	Ubiquinol‐cytochrome c reductase complex assembly factor 6	UQCC6	114.9	86.7	1.33	0.0048	
P56381	ATP synthase subunit epsilon, mitochondrial	ATP5F1E	114.5	87	1.32	0.0076	
J3KS94	Myelin basic protein	MBP	118.1	90.4	1.31	0.0026	
C9JZI7	Nucleosome assembly protein 1 like 4	NAP1L4	113.2	86.9	1.30	0.0087	
Q9BUR5	MICOS complex subunit MIC26	APOO	114.8	88.4	1.30	0.0242	
O75208	Ubiquinone biosynthesis protein COQ9, mitochondrial	COQ9	114.9	88.5	1.30	0.0033	
Q9NRX4	14 kDa phosphohistidine phosphatase	PHPT1	112.0	86.5	1.30	0.0025	
Q15843	NEDD8	NEDD8	114.1	88.2	1.30	0.0176	

For these interacting species, pathway and process enrichment analysis revealed “aerobic respiration”, “cellular respiration”, “oxidative phosphorylation”, and “energy derivation by oxidation of organic compounds” as the four (out of a total of hundred and nineteen) statistically most relevant increased (*p* « 0.01) GO‐terms (Table 10), which came from the protein species ATP synthase subunit epsilon, mitochondrial (ATP5F1E), ATP synthase‐coupling factor 6, mitochondrial (ATP5PF), cytochrome c oxidase subunit 4 isoform 1, mitochondrial (COX4I1), cytochrome c oxidase subunit 5B, mitochondrial (COX5B), cytochrome c oxidase subunit 8A, mitochondrial (COX8A), dihydrolipoyllysine‐residue succinyltransferase component of 2‐oxoglutarate dehydrogenase complex, mitochondrial (DLST), malate dehydrogenase, mitochondrial (MDH2), acyl carrier protein, mitochondrial (NDUFAB1), NADH dehydrogenase [ubiquinone] 1 beta subcomplex subunit 3 (NDUFB3), cytochrome b‐c1 complex subunit 7 (UQCRB), cytochrome c oxidase subunit 5A, mitochondrial (COX5A), ATP synthase subunit d, mitochondrial (ATP5PD), cytochrome c (CYCS), and ubiquinone biosynthesis protein COQ9, mitochondrial (COQ9).

**TABLE 10:** Pathway and process enrichment (GO‐enrichment) analysis applied to the PPI network identified for the over‐represented proteins at t9 + 2 in the comparison “t9 + 2 vs. t11”. Reported are the four most significantly enriched Gene Ontology terms (including GO‐Category, GO‐term identifier, description, involved genes, and *p*‐value).

Category	GO‐term	Description	Genes	*p*‐Value
GO Biological Processes	GO:0009060	Aerobic respiration	ATP5F1E|ATP5PF|COX4I1|COX5B|COX8A|DLST|MDH2|NDUFAB1|NDUFB3|UQCRB|COX5A|ATP5PD|CYCS|COQ9	1E‐22
GO Biological Processes	GO:0045333	Cellular respiration	ATP5F1E|ATP5PF|COX4I1|COX5B|COX8A|DLST|MDH2|NDUFAB1|NDUFB3|UQCRB|COX5A|ATP5PD|CYCS|COQ9	1E‐21
GO Biological Processes	GO:0006119	Oxidative phosphorylation	ATP5F1E|ATP5PF|COX4I1|COX5B|COX8A|NDUFAB1|NDUFB3|UQCRB|COX5A|ATP5PD|CYCS|COQ9	1E‐20
GO Biological Processes	GO:0015980	Energy derivation by oxidation of organic compounds	ATP5F1E|ATP5PF|COX4I1|COX5B|COX8A|DLST|MDH2|NDUFAB1|NDUFB3|UQCRB|COX5A|ATP5PD|CYCS|COQ9	1E‐20

**FIGURE 6:**
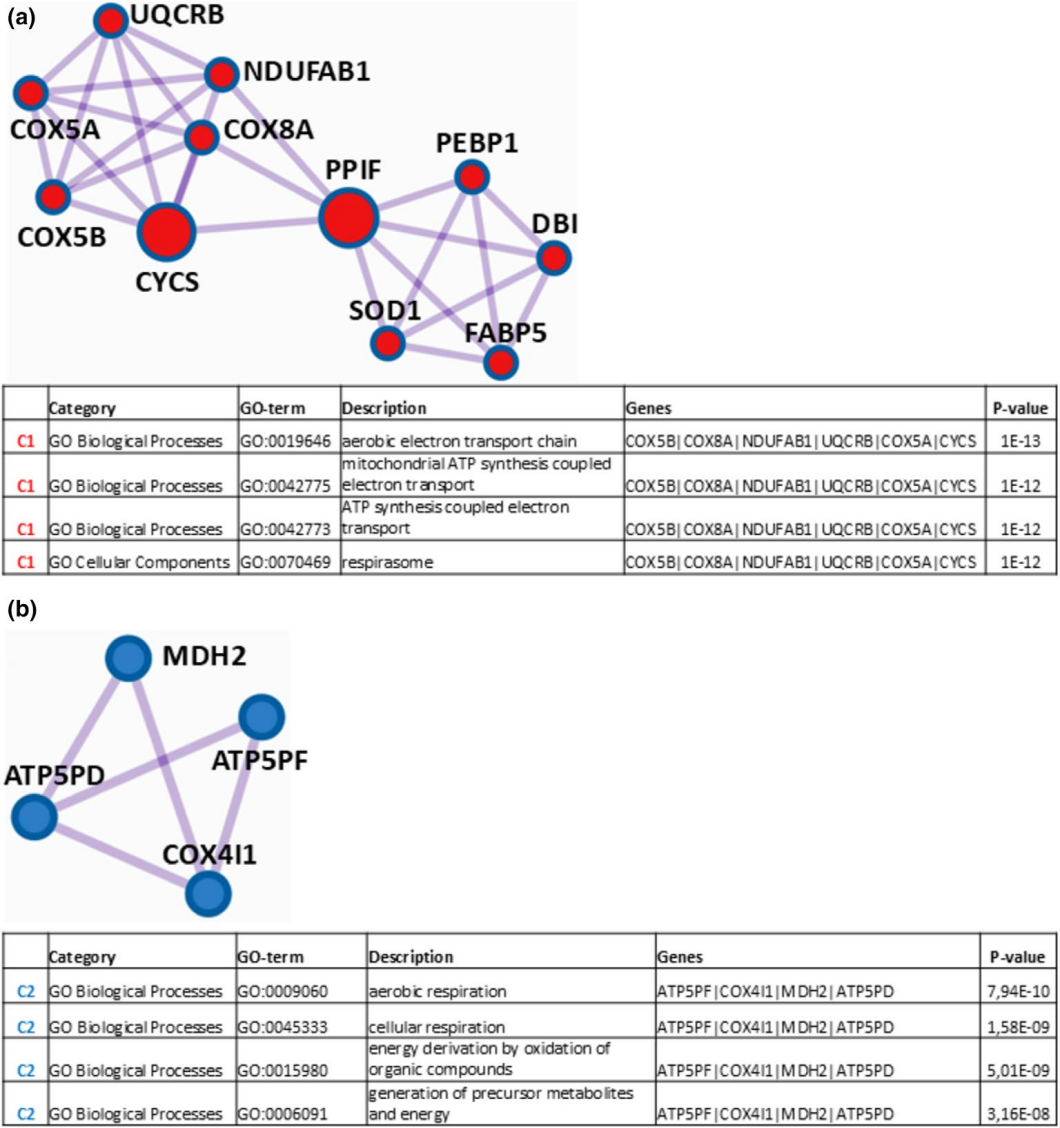
Protein clusters C1 (a) and C2 (b) identified for the over‐represented proteins at t9 + 2 in the comparison “t9 + 2 vs. t11” resulting from Mestascape analysis through the Molecular Complex Detection algorithm and relative GO‐enrichment analysis of the corresponding proteomic results. Shown in the following tables are the four most significantly enriched Gene Ontology terms (including GO‐Category, GO‐term identifier, description, involved genes, and *p*‐value).

Then, Metascape software was able to detect by the MCODE algorithm, within the PPI network, the proteins reported in Figure 6a as strongly connected in the cluster C1, for which pathway and process enrichment analysis highlighted the GO‐terms “aerobic electron transport chain”, “mitochondrial ATP synthesis coupled electron transport”, “ATP synthesis coupled electron transport”, and “respirasome” as the four (out of a total of forty‐one) statistically most significant increased (*p* « 0.01), which arose from the species COX5B, COX8A, NDUFAB1, UQCRB, COX5A and CYCS, as well as the proteins indicated in Figure 6b as strongly interacting in the cluster C2, for which pathway and process enrichment analysis revealed the GO‐terms “aerobic respiration”, “cellular respiration”, “energy derivation by oxidation of organic compounds”, and “generation of precursor metabolites and energy”, as the four (out of a total of 25) statistically most relevant increased (*p* « 0.01), which derived from the species ATP5PF, COX4I1, MDH2, and ATP5PD. These last findings converge towards components/processes associated to cellular respiration.

Consequently, these data revealed that, compared to t11, at t9 + 2 muscle proteome underwent structural/functional modifications distinctly indicating a significant shift associated with a condition characterized by an augmented oxygen consumption. Data here not included for the comparison “t9 + 2 vs. t11”, related to DRPs identification and functional enrichment/PPI network/MCODE analyses, are reported in Table S6.

Concerning the down‐represented proteins at t9 + 2, Metascape software was able to unveil a protein–protein interaction network constituted by the 148 protein species reported in Table 11.

**TABLE 11:** Interacting down‐represented proteins at t9 + 2, as determined by Metascape analysis of results from the comparison “t9 + 2 vs. t11”. Reported are UniProtKB accessions, proteins and corresponding gene names, protein abundances at t9 + 2 (Ab_t9+2_) and t11 (Ab_t11_), abundance ratios Ab_t9+2_/Ab_t11_ (9 + 2/11) and corresponding ratio *p*‐values. Proteins belonging to the clusters (C1, C2, C3, and C4) detected by MCODE algorithm (Figure 7a–d) are also indicated.

UniProtKB Acc.	Protein	Gene	Ab_t9+2_	Ab_t11_	9 + 2/11	*p*‐Value	MCODE
E9PK54	Heat shock cognate 71 kDa protein	HSPA8	90.2	117.7	0.77	0.0301	C1
Q8IWX7	Protein unc‐45 homolog B	UNC45B	88.2	121.0	0.73	0.0025	C1
F5GYJ8	Ubiquitinyl hydrolase 1	OTUB1	86.0	118.9	0.72	0.0029	C1
P11055	Myosin‐3	MYH3	90.7	127.9	0.71	0.0283	C1
A0A5F9ZHM9	Calcium/calmodulin‐dependent protein kinase	CAMK2A	80.3	115.4	0.70	0.0192	C1
C9JUL4	DnaJ heat shock protein family (Hsp40) member B4	DNAJB4	79.2	115.9	0.68	0.0108	C1
O75439	Mitochondrial‐processing peptidase subunit beta	PMPCB	78.8	117.5	0.67	0.028	C1
P17612	cAMP‐dependent protein kinase catalytic subunit alpha	PRKACA	76.1	121	0.63	0.0175	C1
P36507	Dual specificity mitogen‐activated protein kinase kinase 2	MAP2K2	69.1	123.1	0.56	0.0085	C1
A0A2R8Y793	Actin, cytoplasmic 1	ACTB	74.2	147.0	0.50	0.0294	C1
P12882	Myosin‐1	MYH1	66.6	143.3	0.46	0.048	C1
Q562R1	Beta‐Actin‐like protein 2	ACTBL2	65.2	151.5	0.43	0.0074	C1
P0CG38	POTE ankyrin domain family member I	POTEI	55.6	163.0	0.34	0.0427	C1
H7C144	Alpha‐actinin‐4	ACTN4	93.2	123.1	0.76	0.0438	C2
P47755	F‐Actin‐capping protein subunit alpha‐2	CAPZA2	86.9	115.0	0.76	0.0105	C2
K7ELX4	Ferrochelatase	FECH	86.3	116.2	0.74	0.0041	C2
Q16795	NADH dehydrogenase [ubiquinone] 1 alpha subcomplex subunit 9, mitochondrial	NDUFA9	86.1	116.5	0.74	0.032	C2
P34896	Serine hydroxymethyltransferase, cytosolic	SHMT1	84.7	116.8	0.73	0.0237	C2
E5RG95	Enolase 3	ENO3	83.3	116.4	0.72	0.0292	C2
Q8NE86	Calcium uniporter protein, mitochondrial	MCU	86.0	123.1	0.70	0.0063	C2
Q15124	Phosphoglucomutase‐like protein 5	PGM5	83.0	121.2	0.68	0.0244	C2
A0A8V8TR22	Calcium/calmodulin‐dependent protein kinase	CAMK2B	80.2	117.6	0.68	0.0077	C2
Q8IXI1	Mitochondrial Rho GTPase 2	RHOT2	79.9	119.5	0.67	0.0001	C2
A0A8V8TNA1	Calcium/calmodulin‐dependent protein kinase type II subunit delta	CAMK2D	78.1	121.1	0.64	0.0051	C2
Q5SWX3	Calcium/calmodulin‐dependent protein kinase	CAMK2G	74.2	115.3	0.64	0.0219	C2
A0A087WVN4	Farnesyl pyrophosphate synthase	FDPS	79.7	125.8	0.63	0.0017	C2
Q5SV16	ATPase family AAA domain containing 3A	ATAD3A	76.0	122.4	0.62	0.0163	C2
Q16186	Proteasomal ubiquitin receptor ADRM1	ADRM1	85.9	111.4	0.77	0.0148	C3
A0A804HKI2	Leucine rich pentatricopeptide repeat containing	LRPPRC	90.4	117.6	0.77	0.0093	C3
P13489	Ribonuclease inhibitor	RNH1	84.4	109.8	0.77	0.0206	C3
P04843	Dolichyl‐diphosphooligosaccharide‐‐protein glycosyltransferase subunit 1	RPN1	85.6	116.4	0.74	0.0021	C3
P62191	26S proteasome regulatory subunit 4	PSMC1	82.5	113.9	0.72	0.0205	C3
Q9UNM6	26S proteasome non‐ATPase regulatory subunit 13	PSMD13	86.3	119.2	0.72	0.0014	C3
P46199	Translation initiation factor IF‐2, mitochondrial	MTIF2	85.4	118.3	0.72	0.0229	C3
A0A994J6V8	26S proteasome regulatory subunit 8	PSMC5	84.2	116.8	0.72	0.0321	C3
O43242	26S proteasome non‐ATPase regulatory subunit 3	PSMD3	80.4	112.4	0.72	0.0229	C3
E5RIZ4	Large ribosomal subunit protein uL15m	MRPL15	83.7	122.2	0.68	0.0174	C3
O60783	Small ribosomal subunit protein uS14m	MRPS14	81.5	121.7	0.67	0.0286	C3
Q8N163	Cell cycle and apoptosis regulator protein 2	CCAR2	69.2	120.3	0.58	0.0203	C3
P68032	Actin, alpha cardiac muscle 1	ACTC1	35.4	226.7	0.16	0.0011	C3
P50914	Large ribosomal subunit protein eL14	RPL14	87.8	114.5	0.77	0.003	C4
E7EQV9	Ribosomal protein L15	RPL15	85.2	117.0	0.73	0.049	C4
P41227	N‐alpha‐acetyltransferase 10	NAA10	81.5	113.5	0.72	0.0166	C4
K7ERI7	Large ribosomal subunit protein eL22	RPL22	80.4	114.1	0.70	0.0005	C4
G3V3H3	Kinesin light chain	KLC1	80.8	116.2	0.70	0.0135	C4
P61221	ATP‐binding cassette sub‐family E member 1	ABCE1	86.1	125.2	0.69	0.0459	C4
D6RBD0	Small ribosomal subunit protein RACK1	RACK1	83.7	123.1	0.68	0.0024	C4
P19367	Hexokinase‐1	HK1	81.1	122.8	0.66	0.0322	C4
A0A7P0TA76	Long‐chain‐fatty‐acid‐‐CoA ligase	ACSL3	79.9	123.0	0.65	0	C4
O00232	26S proteasome non‐ATPase regulatory subunit 12	PSMD12	71.9	121.7	0.59	0.0133	C4
P17655	Calpain‐2 catalytic subunit	CAPN2	88.5	115.1	0.77	0.0078	
O75396	Vesicle‐trafficking protein SEC22b	SEC22B	87.3	113.6	0.77	0.009	
Q3LXA3	Triokinase/FMN cyclase	TKFC	86.8	113.2	0.77	0.0364	
Q9BXW7	Haloacid dehalogenase‐like hydrolase domain‐containing 5	HDHD5	87.8	114.6	0.77	0.0058	
P63146	Ubiquitin‐conjugating enzyme E2 B	UBE2B	89.4	115.8	0.77	0.0269	
Q6PCB7	Long‐chain fatty acid transport protein 1	SLC27A1	90.3	118.0	0.77	0.0299	
O95336	6‐phosphogluconolactonase	PGLS	88.0	115.3	0.76	0.0149	
P07099	Epoxide hydrolase 1	EPHX1	91.0	119.3	0.76	0.0237	
M0R026	2‐hydroxyacyl‐CoA lyase 2	ILVBL	89.4	117.3	0.76	0.0021	
H9KV45	Ubiquitin‐conjugating enzyme E2 D3	UBE2D3	85.6	112.8	0.76	0.0321	
A0A5F9ZHW5	Ataxin 2	ATXN2	89.7	117.8	0.76	0.0203	
F8VZ52	Dynamin‐1‐like protein	DNM1L	87.9	115.5	0.76	0.0464	
A0A1W2PPS1	Heterogeneous nuclear ribonucleoprotein U	HNRNPU	89.8	118.1	0.76	0.0202	
P46734	Dual specificity mitogen‐activated protein kinase kinase 3	MAP2K3	85.2	112.1	0.76	0.0068	
A0A0A0MTJ9	Neutral cholesterol ester hydrolase 1	NCEH1	86.5	114.1	0.76	0.0188	
Q9NYU2	UDP‐glucose: glycoprotein glucosyltransferase 1	UGGT1	89.2	118.2	0.75	0.0234	
Q8TAE6	Protein phosphatase 1 regulatory subunit 14C	PPP1R14C	86.4	115.4	0.75	0.005	
Q9NTX5	Ethylmalonyl‐CoA decarboxylase	ECHDC1	88.1	117.7	0.75	0.0079	
Q02978	Mitochondrial 2‐oxoglutarate/malate carrier protein	SLC25A11	89.6	119.9	0.75	0.016	
Q9BPX6	Calcium uptake protein 1, mitochondrial	MICU1	85.7	114.9	0.75	0.0169	
Q7L3B6	Hsp90 co‐chaperone Cdc37‐like 1	CDC37L1	86.3	116.1	0.74	0.001	
Q9HA65	TBC1 domain family member 17	TBC1D17	87.1	117.6	0.74	0.0267	
A2A2M0	Regulation of nuclear pre‐mRNA domain‐containing protein	RPRD1B	86.7	117.3	0.74	0.0182	
P36543	V‐type proton ATPase subunit E 1	ATP6V1E1	84.3	114.1	0.74	0.0274	
A1L4K1	Fibronectin type III and SPRY domain‐containing protein 2	FSD2	86.5	117.1	0.74	0.0172	
Q96C86	m7GpppX diphosphatase	DCPS	85.2	115.8	0.74	0.0241	
Q86WU2	Probable D‐lactate dehydrogenase, mitochondrial	LDHD	86.2	117.3	0.73	0.0018	
P07384	Calpain‐1 catalytic subunit	CAPN1	85.1	116.1	0.73	0.0134	
Q9UKU7	Isobutyryl‐CoA dehydrogenase, mitochondrial	ACAD8	86.1	117.6	0.73	0.035	
Q01082	Spectrin beta chain, non‐erythrocytic 1	SPTBN1	85.0	116.8	0.73	0.0246	
Q9H223	EH domain‐containing protein 4	EHD4	87.3	120.0	0.73	0.0308	
Q3ZCQ8	Mitochondrial import inner membrane translocase subunit TIM50	TIMM50	88.4	121.8	0.73	0.0007	
E7ESD2	WASH complex subunit 2A	WASHC2A	85.3	118.0	0.72	0.0276	
Q9Y676	Small ribosomal subunit protein mS40	MRPS18B	83.1	115.2	0.72	0.0458	
A0A994J4Y8	Serine/threonine‐protein phosphatase PGAM5, mitochondrial	PGAM5	85.7	118.9	0.72	0.0224	
M0R1F3	DM1 protein kinase	DMPK	82.7	114.8	0.72	0.0231	
E9PLK3	Aminopeptidase	NPEPPS	85.4	118.8	0.72	0.0126	
Q15172	Serine/threonine‐protein phosphatase 2A 56 kDa regulatory subunit alpha isoform	PPP2R5A	83.5	116.4	0.72	0.0053	
Q9Y613	FH1/FH2 domain‐containing protein 1	FHOD1	82.6	115.2	0.72	0.0247	
A0A8V8TQK0	WD repeat domain 1	WDR1	81.2	113.6	0.71	0.0074	
E5RIM3	Phospholipase A2 activating protein	PLAA	84.5	118.3	0.71	0.0035	
P48449	Lanosterol synthase	LSS	91.2	128.3	0.71	0.029	
Q99961	Endophilin‐A2	SH3GL1	88.3	124.3	0.71	0.0439	
G3V180	Dipeptidyl peptidase 3	DPP3	85.3	120.1	0.71	0.016	
P53004	Biliverdin reductase A	BLVRA	78.9	111.1	0.71	0.0258	
Q15102	Platelet‐activating factor acetylhydrolase IB subunit alpha1	PAFAH1B3	85.4	120.7	0.71	0.0154	
E5RIA4	Aspartyl aminopeptidase	DNPEP	83.3	117.9	0.71	0.0064	
Q03113	Guanine nucleotide‐binding protein subunit alpha‐12	GNA12	81.1	115.2	0.70	0.0286	
A0A7I2V2S3	Exportin 1	XPO1	85.0	121.1	0.70	0.0174	
F8VZE0	Myosin binding protein C1	MYBPC1	81.7	117.4	0.70	0.0016	
A0A994J4W8	Ankyrin 1	ANK1	80.3	115.4	0.70	0.0328	
O15239	NADH dehydrogenase [ubiquinone] 1 alpha subcomplex subunit 1	NDUFA1	83.1	119.7	0.69	0.014	
Q8NCW5	NAD(P)H‐hydrate epimerase	NAXE	80.1	115.3	0.69	0.009	
P14868	Aspartate‐‐tRNA ligase, cytoplasmic	DARS1	86.1	124.0	0.69	0.0036	
O75746	Electrogenic aspartate/glutamate antiporter SLC25A12, mitochondrial	SLC25A12	85.3	123.0	0.69	0.0048	
Q96ER9	Mitochondrial potassium channel	CCDC51	87.1	125.6	0.69	0.0456	
J3KN42	Solute carrier family 25 member 24	SLC25A24	83.4	121.1	0.69	0.0014	
Q92665	Small ribosomal subunit protein mS31	MRPS31	79.6	116.3	0.68	0.0135	
Q13203	Myosin‐binding protein H	MYBPH	81.3	118.9	0.68	0.0309	
Q9BXI3	Cytosolic 5′‐nucleotidase 1A	NT5C1A	83.8	122.6	0.68	0.0008	
A0A0B4J1R2	Acyl‐CoA synthetase short‐chain family member 3, mitochondrial	ACSS3	77.1	113.0	0.68	0.0154	
Q0ZGT2	Nexilin	NEXN	80.4	118.0	0.68	0.0433	
Q13098	COP9 signalosome complex subunit 1	GPS1	84.7	124.5	0.68	0.0033	
P13010	X‐ray repair cross‐complementing protein 5	XRCC5	76.1	111.9	0.68	0.0424	
Q9Y696	Chloride intracellular channel protein 4	CLIC4	80.2	118.1	0.68	0.0132	
Q12931	Heat shock protein 75 kDa, mitochondrial	TRAP1	83.3	122.9	0.68	0.0083	
O60763	General vesicular transport factor p115	USO1	76.7	113.2	0.68	0.0488	
Q13619	Cullin‐4A	CUL4A	80.6	119.6	0.67	0.0046	
Q86TU7	Actin‐histidine N‐methyltransferase	SETD3	80.0	119.1	0.67	0.0035	
A0A024R571	EH domain containing 1	EHD1	82.7	123.5	0.67	0.0204	
Q13976	cGMP‐dependent protein kinase 1	PRKG1	81.9	123.0	0.67	0.0081	
A0A6Q8PFJ4	Mitofusin 2	MFN2	84.7	128.2	0.66	0.0149	
Q8N1G4	Leucine‐rich repeat‐containing protein 47	LRRC47	77.7	118.1	0.66	0.0058	
F8VQX6	Thiol methyltransferase 1A	TMT1A	78.2	120.2	0.65	0.0074	
O95168	NADH dehydrogenase [ubiquinone] 1 beta subcomplex subunit 4	NDUFB4	85.0	130.3	0.65	0.0289	
A0A994J4T3	Ubiquitinyl hydrolase 1	USP9X	77.3	118.4	0.65	0.0071	
E9PHT6	4′‐phosphopantetheine phosphatase	PANK4	77.6	119.7	0.65	0.0123	
A0A3B3IT39	Selenoprotein F	SELENOF	76.2	118.3	0.64	0.0191	
Q5VY30	Retinol‐binding protein	RBP4	75.0	116.7	0.64	0.0273	
P17980	26S proteasome regulatory subunit 6A	PSMC3	76.8	120.2	0.64	0.0172	
P04844	Dolichyl‐diphosphooligosaccharide‐‐protein glycosyltransferase subunit 2	RPN2	80.6	126.8	0.64	0.0065	
P68104	Elongation factor 1‐alpha 1	EEF1A1	81.8	129.4	0.63	0.0178	
P16284	Platelet endothelial cell adhesion molecule	PECAM1	79.7	126.4	0.63	0.0341	
A0A7I2V381	Insulin like growth factor 2 receptor	IGF2R	77.9	123.7	0.63	0.0079	
Q9P032	NADH dehydrogenase [ubiquinone] 1 alpha subcomplex assembly factor 4	NDUFAF4	74.9	118.7	0.63	0.0031	
P21817	Ryanodine receptor 1	RYR1	74.8	119.3	0.63	0.0187	
A0A7I2V2U8	Twinfilin Actin binding protein 2	TWF2	81.5	130.2	0.63	0.0042	
P04004	Vitronectin	VTN	80.0	127.9	0.63	0.0205	
A0A8Q3WKN4	Complement C8 alpha chain	C8A	79.5	127.6	0.62	0.0391	
O94905	Erlin‐2	ERLIN2	72.8	120.2	0.61	0.0477	
Q9Y6P5	Sestrin‐1	SESN1	71.5	118.4	0.60	0.0108	
P11277	Spectrin beta chain, erythrocytic	SPTB	76.8	129.6	0.59	0.0215	
Q12904	Aminoacyl tRNA synthase complex‐interacting multifunctional protein 1	AIMP1	72.6	123.8	0.59	0.0071	
E9PB90	Hexokinase‐2	HK2	75.3	131.5	0.57	0.0002	
Q14324	Myosin‐binding protein C, fast‐type	MYBPC2	73.9	131.5	0.56	0.0497	
F5H563	Erythrocyte membrane protein band 4.2	EPB42	71.4	137.1	0.52	0.0053	
Q96LW7	Caspase recruitment domain‐containing protein 19	CARD19	72.0	138.4	0.52	0.0133	
A5A3E0	POTE ankyrin domain family member F	POTEF	59.7	171.7	0.35	0.004	

Metascape pathway and process enrichment analysis ran for these interacting proteins revealed “mitochondrial membrane”, “myofibril”, “organelle inner membrane”, and “mitochondrial inner membrane” as the four (out of a total of four hundred and sixty‐two) statistically most significant decreased (*p* « 0.01) GO‐terms (Table 12), which resulted not very informative about the biological meaning linked to the decreasing proteins at t9 + 2 with respect to the t11 condition.

**TABLE 12:** Pathway and process enrichment (GO‐enrichment) analysis applied to the PPI network identified for the down‐represented proteins at t9 + 2 in the comparison “t9 + 2 vs. t11”. Reported are the four most significantly enriched Gene Ontology terms (including GO‐Category, GO‐term identifier, description, involved genes, and *p*‐value).

Category	GO‐term	Description	Genes	*p*‐Value
GO Cellular Components	GO:0031966	Mitochondrial membrane	DMPK|ACSL3|FECH|HK1|HK2|NDUFA1|NDUFA9|NDUFB4|SLC25A11|SLC25A12|PMPCB|MFN2|DNM1L|TRAP1|MRPS31|MICU1|MRPS18B|NDUFAF4|MRPL15|SLC25A24|ATAD3A|MRPS14|CCDC51|CARD19|RHOT2|MCU|TIMM50|PGAM5|LDHD|SLC27A1	1E‐18
GO Cellular Components	GO:0030016	Myofibril	ACTC1|ACTN4|ANK1|MYBPC1|MYBPC2|MYBPH|MYH1|MYH3|PGM5|PPP2R5A|RPL15|RYR1|SPTBN1|DNAJB4|TWF2|NEXN|UNC45B	1E‐14
GO Cellular Components	GO:0019866	Organelle inner membrane	FECH|NDUFA1|NDUFA9|NDUFB4|SLC25A11|SLC25A12|PMPCB|LRPPRC|TRAP1|MRPS31|MICU1|MRPS18B|NDUFAF4|MRPL15|SLC25A24|ATAD3A|MRPS14|CCDC51|MCU|TIMM50|PGAM5|LDHD|SLC27A1	1E‐14
GO Cellular Components	GO:0005743	Mitochondrial inner membrane	FECH|NDUFA1|NDUFA9|NDUFB4|SLC25A11|SLC25A12|PMPCB|TRAP1|MRPS31|MICU1|MRPS18B|NDUFAF4|MRPL15|SLC25A24|ATAD3A|MRPS14|CCDC51|MCU|TIMM50|PGAM5|LDHD|SLC27A1	1E‐14

**FIGURE 7:**
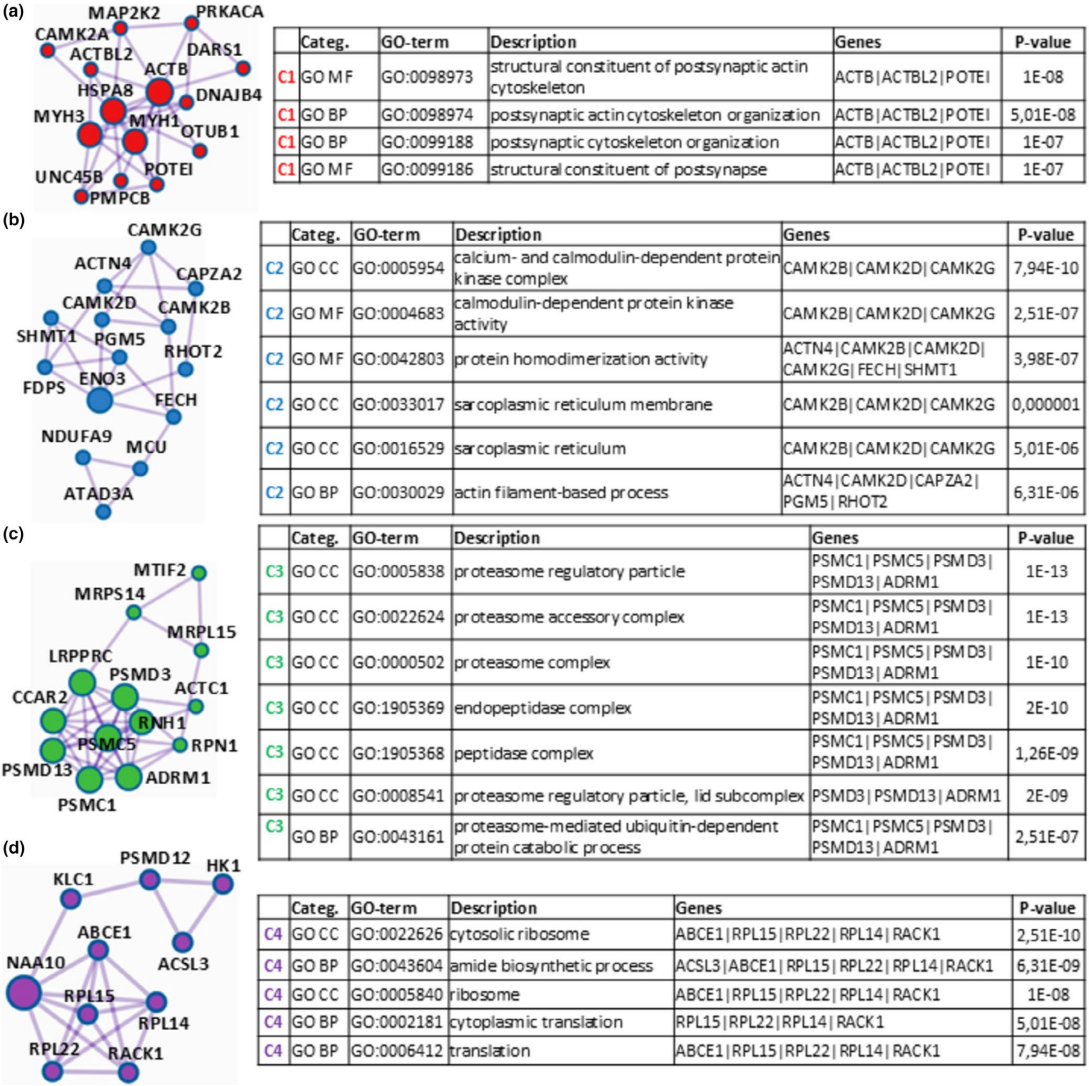
Protein clusters (C1 (a), C2 (b), C3 (c), and C4 (d)) identified for the down‐represented proteins at t9 + 2 in the comparison “t9 + 2 vs. t11” resulting from Mestascape analysis through the Molecular Complex Detection algorithm and relative GO‐enrichment analysis of the corresponding proteomic results. Shown in the following tables are the most significantly enriched Gene Ontology terms (including GO‐Category, GO‐term identifier, description, involved genes, and *p*‐value).

To go deep into the functional significance of proteomic data, Metascape software operated the MCODE algorithm to the ascertained PPI network and was able to extract the four protein clusters reported in Figure 7 constituted by strongly connected proteins, also indicated in Table 11 (column MCODE), for each of which pathway and process enrichment analysis was performed. For cluster C1 (Figure 7a), this analysis identified “structural constituent of postsynaptic actin cytoskeleton”, “postsynaptic actin cytoskeleton organization”, “postsynaptic cytoskeleton organization”, and “structural constituent of postsynapse” as the four (out of a total of 67) statistically most relevant decreased (*p* « 0.01) GO‐terms, arising from actin, cytoplasmic 1 (ACTB), beta‐actin‐like protein 2 (ACTBL2), and POTE ankyrin domain family member I (POTEI). For cluster C2 (Figure 7b), it revealed the GO‐terms “calcium‐ and calmodulin‐dependent protein kinase complex”, “calmodulin‐dependent protein kinase activity”, “protein homodimerization activity”, “sarcoplasmic reticulum membrane”, “sarcoplasmic reticulum”, and “actin filament‐based process” as the six (out of a total of forty‐one) statistically most significant decreased (*p* « 0.01), coming from calcium/calmodulin‐dependent protein kinase (CAMK2B), calcium/calmodulin‐dependent protein kinase type II subunit delta (CAMK2D), calcium/calmodulin‐dependent protein kinase (CAMK2G), alpha‐actinin‐4 (ACTN4), ferrochetalase (FECH), serine hydroxymethyltransferase, cytosolic (SHMT1), F‐actin‐capping protein subunit alpha‐2 (CAPZA2), phosphoglucomutase‐like protein 5 (PGM5), and mitochondrial Rho GTPase 2 (RHOT2). For cluster C3 (Figure 7c), the GO‐terms “proteasome regulatory particle”, “proteasome accessory complex”, “proteasome complex”, “endopeptidase complex”, “peptidase complex”, “proteasome regulatory particle, lid subcomplex”, and “proteasome‐mediated ubiquitin‐dependent protein catabolic process” were detected as the seven (out of a total of forty‐one) statistically most significant decreased (*p* « 0.01), deriving from 26S proteasome regulatory subunit 4 (PSMC1), 26S proteasome regulatory subunit 8 (PSMC5), 26S proteasome non‐ATPase regulatory subunit 3 (PSMD3), 26S proteasome non‐ATPase regulatory subunit 13 (PSMD13), and proteasomal ubiquitin receptor ADRM1 (ADRM1). For cluster C4 (Figure 7d), this analysis identified the GO‐terms “cytosolic ribosome”, “amide biosynthetic process”, “ribosome”, “cytoplasmic translation”, and “translation” as the five (out of a total of seventeen) statistically most significant decreased (*p* « 0.01), originating from ATP‐binding cassette sub‐family E member 1 (ABCE1), large ribosomal subunit protein uL15m (RPL15), large ribosomal subunit protein eL22 (RPL22), large ribosomal subunit protein eL14 (RPL14), small ribosomal subunit protein RACK1 (RACK1) and long‐chain‐fatty‐acid‐‐CoA ligase (ACSL3). Table S6 reports all information here not included for the comparison “t9 + 2 vs. t11”, related to DRPs identification and functional enrichment/PPI network/MCODE analyses.

Summarizing, these last results clearly highlighted muscle proteome structural/functional changes at t9 + 2 with respect to t11, which were characterized by a decrease in cellular components/processes associated with muscle structure (as proven by GO‐terms linked to actin cytoskeleton/sarcoplasmic reticulum and their organization, enriched for cluster C1 and C2), by a diminished protein synthesis (as demonstrated by GO‐terms linked to ribosome/translation, enriched for cluster C4) and, finally, by a reduction of protein recycle (as established by GO‐terms linked to proteasome/protein catabolism, enriched for cluster C3).

**FIGURE 8:**
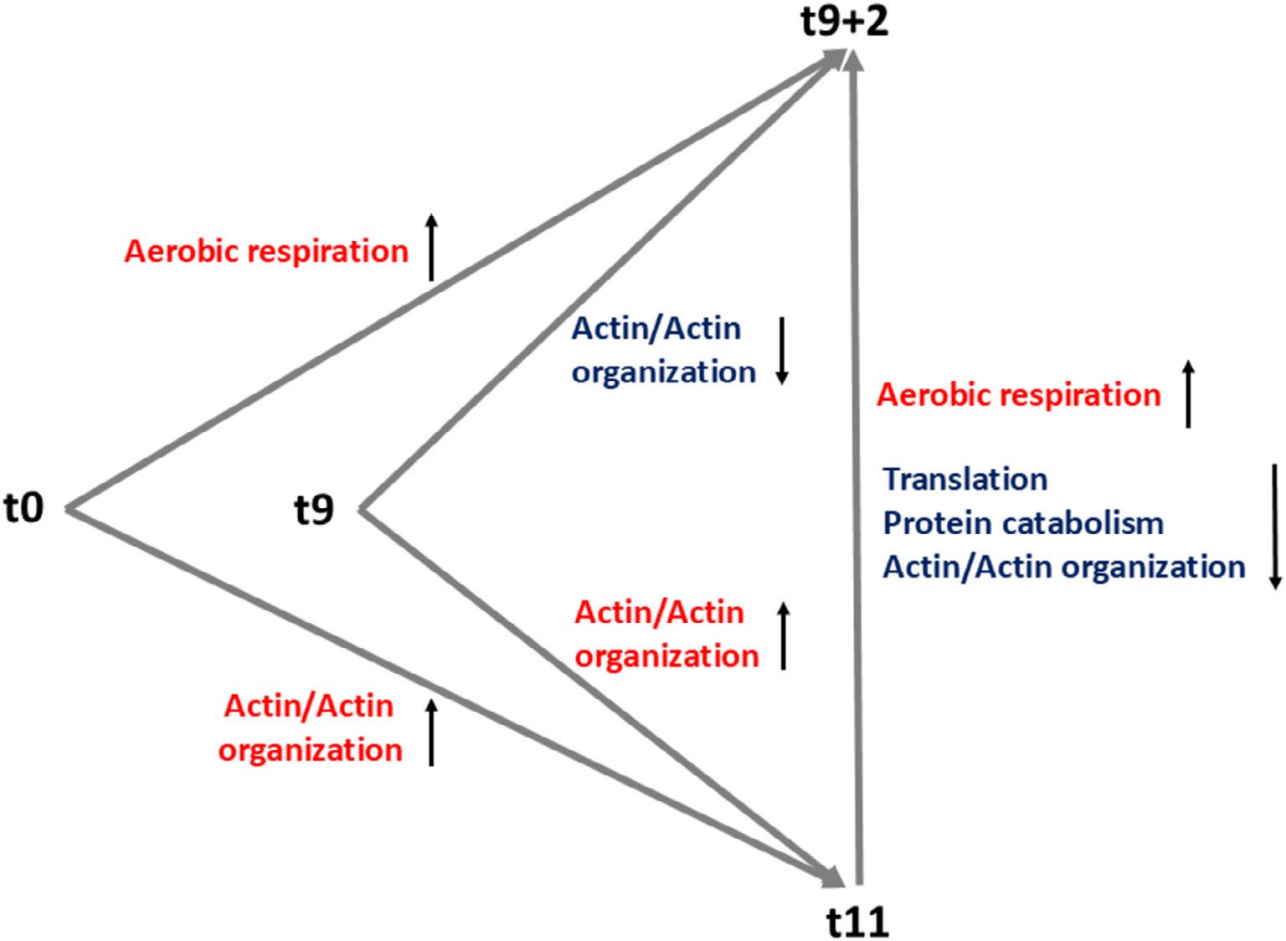
Principal proteomic results based on the binary comparisons described in the present study.

An overview of the proteomic results of this study is given in Figure 8.

### 3.4 Comparison of fiber‐type ratios, capillary density, and cross‐sectional areas at t0, t9, and t11/t9 + 2

**FIGURE 9:**
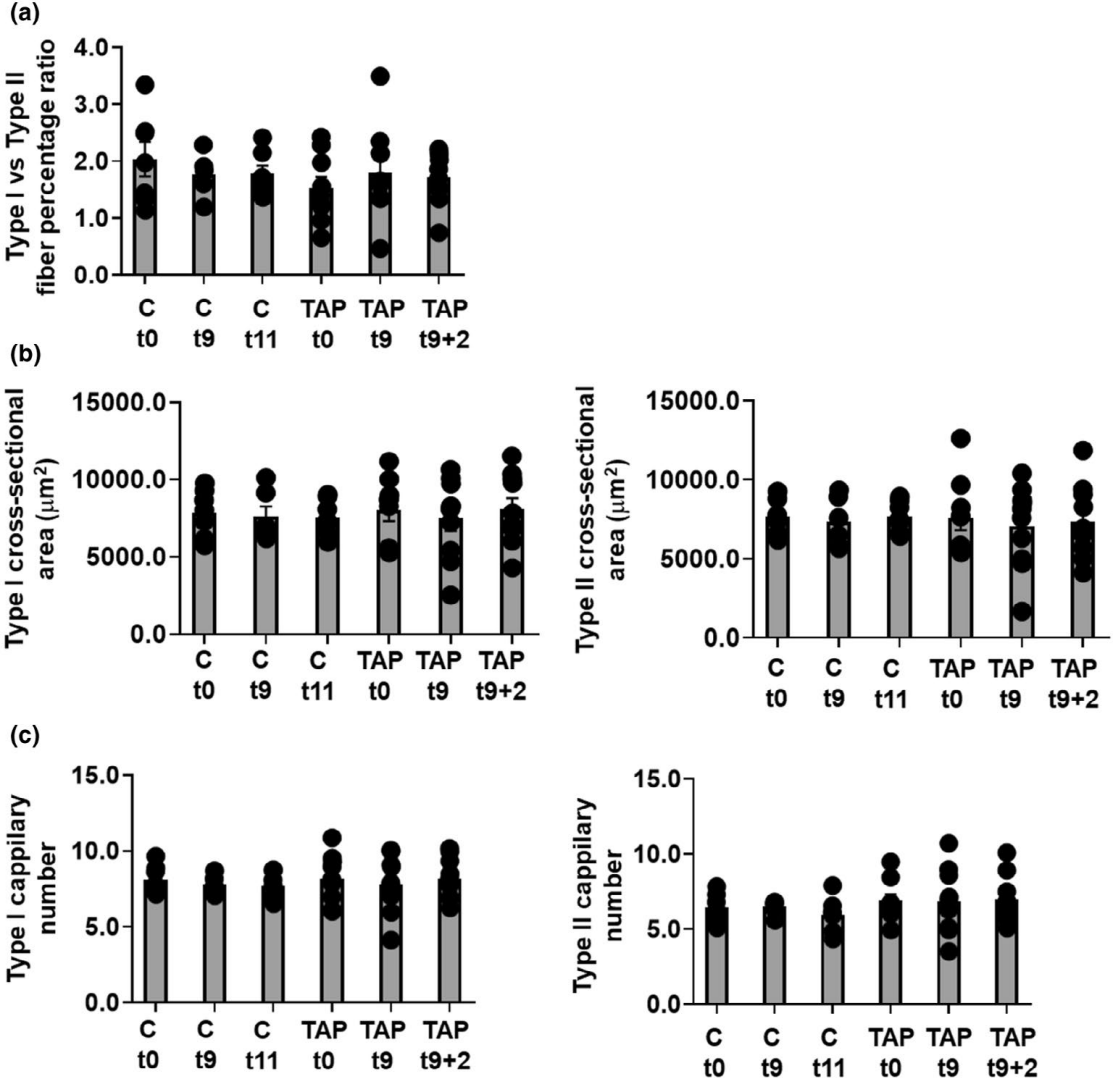
Fiber analysis. (a) Type I /Type II fiber percentage ratios, (b) Fiber‐specific cross‐section areas (μM), and (c) Capillary number per fiber throughout the intervention period between the tapering and the control group. TAP, taper.

Finally, we assessed whether the tapering phase in the studied participants caused a change in fiber composition in the corresponding *vastus lateralis* muscle. We did not observe between‐group changes in the Type I/Type II fiber ratios throughout the intervention (see Figure 9a). Moreover, no changes in fiber type specific cross‐sectional areas (Figure 9b) and capillary density (Figure 9c) were observed between time and groups in the control and volume tapering group.

## 4 DISCUSSION

Alleviation of training protocols has been shown to differentially affect performance (Bosquet et al., 2007; García‐Pallarés et al., 2009; Madsen et al., 1993; Mujika, 2010; Mujika & Padilla, 2000; Pedlar et al., 2018; Spiering et al., 2021). Complete cessation of training has been shown to decrease maximal oxygen uptake (V̇O2max) by 4%–14% in (García‐Pallarés et al., 2009; Mujika & Padilla, 2000), and short‐term reductions in training load (<4 weeks) could already lead to performance impairments (García‐Pallarés et al., 2009; Mujika & Padilla, 2000; Pedlar et al., 2018). The reasons for these discrepancies are up to now unknown, and apart from studies on selected mitochondrial proteins and genes involved in proteolysis and myogenesis, no data are available as to how overall protein profiles in muscle underlie the observed outcomes. Therefore, the aim of this study was to investigate the effect of a two‐week, 50% training volume reduction on cycling performance (tapering phase) after a nine‐week training period in well‐trained cyclists, and to compare the outcomes with the corresponding proteomic and fiber profiles of *vastus lateralis* muscle. Similar to what has been applied in previous studies (Granata et al., 2016; Luden et al., 2010), training intensity was kept constant.

At the physiological level, we found that overall, maximal aerobic and anaerobic performance parameters were not affected with respect to controls. After 9 weeks of training, athletes did not yet reach a sufficient cumulation of training load, as confirmed by CON, which continued to improve in their power at LT1 and LT2 throughout the last 2 weeks of training, while TAPER had a significantly lower threshold power, which, however, did not decrease with respect to the 9‐week period. A loss of power at LT2 is not desired, as various studies have shown this to be a powerful predictor for cycling performance (Faude et al., 2009; Heuberger et al., 2018). Contrarily, the main results at the protein level were that with respect to the fully trained group (t11) the taper group (t9 + 2) was characterized by an increased amount of proteins that determine structural and functional aspects of the mitochondrial machinery, including those forming the mitochondrial respiratory chain complex IV, cytochrome‐c oxidase activity, and the rotational mechanism of the proton‐transporting ATP synthase. In addition, a functional protein cluster related to aerobic respiration and oxidative processes was increased in the taper group (t9 + 2). This means that the taper phase, whilst not decreasing submaximal performance, increases the oxidative capacity of muscle. Interestingly, in order to maintain and establish a high level of aerobic capacity, high training volumes are generally necessary in trained athletes (Granata et al., 2016; Seiler, 2010); a possible alternative may be a short‐term reduction of training volume, preparing the muscle tissue to enhance oxygen consumption. Indeed, mitochondrial density regulates substrate metabolism and, therefore, lactate production during submaximal exercise, and an increase has been associated with training intensity (Egan & Zierath, 2013). Changes in muscle fiber type distribution and myosin ATPase expression have been shown to be associated with changes in oxidative capacity and could, therefore, possibly explain changes in lactate thresholds (Egan & Zierath, 2013; Jaspers et al., 2017; Yan et al., 2011). Although at t9 + 2 versus t11 we have observed decreases in the abundance of both Myosin heavy chain 1 (MYH1 or myosin‐1) and Myosin heavy chain 3 (MYH3 or myosin‐3), which has been shown to regulate fiber type ratios towards Type I fibers in pig skeletal muscle (Cho et al., 2019), we did not observe changes in the fiber type I/II ratios in response to tapering, since at t9 + 2 and t11 these ratios were similar.

Rietjens and colleagues found a maintained V̇O_2_max and peak power output over 21 days of training volume and intensity reduction in cyclists (Rietjens et al., 2001). More specifically, a 50% training volume reduction together with either a training intensity of 68% or 83% V̇O_2_max did not affect performance values. These findings are similar to the data observed in the present study, where solely training volume was reduced by ~50%, showing that maximal performance parameters such as V̇O_2_max and PPO_R_ are likely to be unaffected by short periods of tapering. However, in the present investigation, training intensity was maintained, while it was reduced in the study by Rietjens and coworkers.

Interestingly, in one study, high‐intensity‐interval cycling increased mitochondrial protein content and that of mitochondrial transcription factor A (TFAM), nuclear respiratory factor (NRF)1, and Peroxisome proliferator‐activated receptor‐gamma coactivator (PGC)‐1α, which were normalized to control levels after a reduction in training volume (Granata et al., 2016). A second study reported reduced atrogin‐1, MuRF‐1, MRF4, and myostatin gene expression in response to a halving of exercise volume during running (Luden et al., 2010). These findings are in apparent contrast to ours, indicating increased mitochondrial structural proteins and those related to aerobic respiration as a consequence of the tapering phase. This may be related to differences in the training volumes and different distributions of training volume and intensity over the intervention period, which by themselves cause increased content of factors involved in oxidative metabolism (Granata et al., 2016; Luden et al., 2010), whereas the contrary occurs in the intervention described in this study. Of note, it has been observed that muscle may respond better to exercise after tapering on the basis of ameliorated single muscle fiber strength and contraction in runners (Trappe et al., 2000). In addition, a swim taper study showed increased power in type II muscle fibers in response to the tapering intervention, with type II fibers having a larger diameter (Harber et al., 2004). In light of the apparent discrepancies in the outcomes found between different protocols, it becomes increasingly clear that further research is needed to investigate the interplay of training intensity and volume for maintaining performance in general and moreover over short tapering periods, which may increase our insight into how subtle changes in training volume and intensity may affect structural and functional aspects of muscle, including that of proteins involved in oxygen consumption and muscle fibers.

One weakness of the study is that although the proteome profile indicates a shift towards decreased strength and increased aerobic respiration in the *vastus lateralis* muscle as an effect of the TAPER intervention, no strength measurements were completed, which only allows a speculation on the correlation between the protein profile and strength. A second weakness of this protocol is that since athletes performed training individually and received feedback, the individual training volumes may partly explain the heterogenous responses in performance parameters. However, while this may be considered a weakness, separation into the two groups was performed at t9 based on the actual weekly training volume that was achieved. As such, the groups were well‐balanced, and when correlating performance parameters with mean weekly training volume, no significant associations could be found.

In conclusion, a training volume reduction of ~50% during cycling maintains most performance adaptations; however, submaximal performance may be affected. Based on the literature and the given results, the type of response may be linked to the initial training volume, intensity, volume at different intensities, and time of the training period. This underlines the potential of short‐time interventions to induce changes in muscle protein profiles.

## Supporting information


Table S1.

Table S2.

Table S3.

Table S4.

Table S5.

Table S6.

Table S7.
