# Supplementary material for: Unaltered maximal power and submaximal performance correlates with an oxidative vastus lateralis proteome phenotype during tapering in male cyclists
Source: Physiol Rep. 2025 Apr 23;13(8):e70302. doi: 10.14814/phy2.70302 (PMC12015642; doi:10.14814/phy2.70302)
Supplement: Supplementary file 7 — Table S7. [file PHY2-13-e70302-s003.docx]

Mean weekly TSS and luTRIMP are displayed in Table 3. TSS and luTRIMP did not statistically differ between groups apart from training phase IV. During this phase, TSS and luTRIMP were statistically higher in the control group compared to the deloading group (p = 0.013 and p = 0.029, respectively).

Table S7: Mean weekly training stress score (TSS) and Lucias training impulse (luTRIMP) per training phase for deloading and control group.

|  | **TSS** |  |  | **luTRIMP** |  |  |
| --- | --- | --- | --- | --- | --- | --- |
|  | **Deloading** | **Control** | **Between-group p** | **Deloading** | **Control** | **Between-group p** |
| Training Phase I | 95±26 | 115±21 | 0.168 | 151±29 | 177±32 | 0.161 |
| Training Phase II | 99±16 | 145±50 | 0.076 | 171±44 | 210±53 | 0.182 |
| Training Phase III | 182±65 | 163±41 | 0.532 | 208±74 | 212±49 | 0.914 |
| Training Phase IV | 109±23 | 175±50 | 0.013 | 141±57 | 239±80 | 0.029 |
